# Glucosinolate Profile and Glucosinolate Biosynthesis and Breakdown Gene Expression Manifested by Black Rot Disease Infection in Cabbage

**DOI:** 10.3390/plants9091121

**Published:** 2020-08-30

**Authors:** Mehede Hassan Rubel, Md. Abuyusuf, Ujjal Kumar Nath, Arif Hasan Khan Robin, Hee Jeong Jung, Hoy Taek Kim, Jong In Park, Ill Sup Nou

**Affiliations:** 1 Department of Horticulture, Sunchon National University, 255, Jungang-ro, Suncheon, Jeonnam 57922, Korea; mehede@nstu.edu.bd (M.H.R.); yusuf_agr@pstu.ac.bd (M.A.); gml79wjd@sunchon.ac.kr (H.J.J.); htkim@sunchon.ac.kr (H.T.K.); 2 Department of Genetics and Plant Breeding, Bangladesh Agricultural University, Mymensingh 2202, Bangladesh; ujjalnath@gmail.com (U.K.N.); gpb21bau@gmail.com (A.H.K.R.)

**Keywords:** glucosinolate profile, gene expression, *Xcc*, black rot, cabbage

## Abstract

Cabbage (*Brassica oleracea* var. *capitata*) is an economically important crop in the family Brassicaceae. Black rot disease is a top ranked cabbage disease, which is caused by *Xanthomonas campestris* pv. *campestris* (*Xcc*) and may reduce 50% crop loss. Therefore, we need a clear understanding of black rot disease resistance for sustainable disease management. The secondary metabolites, like Glucosinolate (GSL) presents in *Brassica* species, which plays a potential role in the defense mechanism against pathogens. However, there is little known about GSL-regulated resistance mechanisms and GSL biosynthesis and the breakdown related gene expression after black rot disease infection in cabbage. In this study, relative expression of 43 biosynthetic and breakdown related GSLs were estimated in the black rot resistant and susceptible cabbage lines after *Xcc* inoculation. Ten different types of GSL from both aliphatic and indolic groups were identified in the contrasting cabbage lines by HPLC analysis, which included six aliphatic and four indolic compounds. In the resistant line, nine genes (*MYB122-Bol026204*, *MYB34-Bol017062*, *AOP2-Bo9g006240*, *ST5c-Bol030757*, *CYP81F1-Bol017376*, *CYP81F2-Bol012237*, *CYP81F4-Bol032712*, *CYP81F4-Bol032714* and *PEN2-Bol030092*) showed consistent expression patterns. Pearson’s correlation coefficient showed positive and significant association between aliphatic GSL compounds and expression values of *ST5c-Bol030757* and *AOP2-Bo9g006240* genes as well as between indolic GSL compounds and the expression of *MYB34-Bol017062*, *MYB122-Bol026204*, *CYP81F2-Bol012237*, *CYP81F4-Bol032712* and *CYP81F4-Bol032714* genes. This study helps in understanding the role of GSL biosynthesis and breakdown related genes for resistance against black rot pathogen in cabbage, which could be further confirmed through functional characterization either by overexpression or knock-out mutation.

## 1. Introduction

The bacterial species *Xanthomonas campestris* infects a wide range of *Brassica* species, including cabbage. Korea is among the larger cabbage producers and stands on fifth position in the world, worth approximately 66 million US$ from 2.12 million ton per year [1]. Black rot is a major disease in cabbage and was first identified in Korea in the 1970s [2]. It is a seed-borne disease and is caused by necrotrophic plant bacteria *Xanthomonas campestris* pv. *campestris* (*Xcc*) [3]. This pathogen is distributed frequently around the world and causes 50% economic loss [4]. The *Xcc* pathogen invades the xylem tissues after penetrating through hydathodes and wounded tissues, afterwards disease symptoms are developed in the host plants at warm and humid conditions [5]. Eleven races of the *Xcc* pathogen have been reported, which are proposed based on interaction between *Xcc* strains and differential *Brassica* cultivars following the gene-for-gene model [6,7]. Among them, races 1 and 4 were reported as the most virulent in cabbage [8]. Black rot disease management was attempted by using pathogen free seeds in cultivation, elimination of infected plant debris and cruciferous weeds, crop rotation and finally applying agrochemicals [9]. However, the use of resistant varieties at the farmers level will be the most sustainable way to control the disease [10]. 

Different defense reactions of host-pathogen such as resistance, susceptibility or lack of interaction in host plants are induced by the pathogen [11]. Glucosinolates (GSLs) were reported to show functions in plant defense mechanisms against pathogens [12,13]. GSLs are sulfur and nitrogen containing secondary metabolites broadly found in different *Brassica* species. They could be conferred as resistant against pathogens and insects [14]. Two important types of GSLs (aliphatic and indolic) are predominant in the crops of Brassicaceae and *Arabidopsis* [15,16]. GSLs are hydrolyzed by an endogenous myrosinase enzyme (*β*-thioglucoside glucohydrolase; EC 3.2.1.147) in ruptured cells and most of the hydrolyzed products seem to act as key regulators in tolerance reactions against *Xcc* pathogens [11,17]. The GSL hydrolyzed products, especially isothiocyanates (ITCs) are reported to have beneficial effects, like anti-carcinogenic and anti-oxidative activities for reducing risk of degenerative diseases in human health [18,19]. 

GSL content is varied within *Brassica* species or between cultivars due to allelic variation of GSL biosynthesis genes [20]. It has been noted that three loci (*GSL-PRO*, *GSL-ELONG* and *GSL-ALK*) largely regulate the aliphatic GSL profile in *B. oleracea* [21]. However, the GSL profile might be altered or manifested by pathogenic infections in plants. Many reports have concluded that resistance to necrotrophs, biotrophs and hemibiotrophs are related to indolic GSLs content in *Brassica* crops [22]. In kale, two types of aliphatic GSLs (sinigrin and glucoiberin) and one indolic GSL (glucobrassicin) in leaves are modulated upon *Xcc* infection [23]. In *Arabidopsis*, GSL is hydrolyzed by a myrosinase gene, *PENETRATION2* (*PEN2*), and accumulated as indolic 4-methoxy-glucobrassicin in cells upon expression of *CYP81F2* gene in response against fungal pathogen infection [24]. 

A number of studies (in vivo and in vitro) have been conducted to evaluate the effects of GSLs and their hydrolyzed products on disease development in *Brassica* crops. The transgenic *A. thaliana* with an enhanced amount of individual GSL compound showed resistance against *Erwinia carotovora* and *Pseudomonas syringae* pv. *maculicola* [25]. In *B. napus*, cultivars with higher GSLs content showed resistance against fungal pathogens (*Alternaria* spp. and *Leptosphaeria maculans*) compared to the cultivars with low GSLs [26]. The ITCs are produced upon GSL breakdown and showed biocidal effects in reducing soil-borne plant pathogens [27,28]. GSL-hydrolyzed products (GHP) were found as effective to control several plant pathogenic bacteria, such as *Erwinia chrysanthemi*, *Agrobacterium tumefaciens*, *Pseudomonas tomato*, *Pseudomonas cichorii*, *X. juglandis*, *X. campestris* [11]. Recently, a study on the interaction of kale and *Xcc* pathogen suggested that indolic GSL compound (glucobrassicin) is more effective in decreasing disease severity than aliphatic GSL compounds [23]. 

Management of black rot disease is very difficult because of the wide host range of the pathogens. Therefore, secondary metabolites could be used as an alternate biocide for sustainable disease management in *Brassica* crops [25,29]. To date, only few works have been reported that assess the role of GSLs and their derived products against *Xcc* pathogen in cabbage. The effects of GSLs, ITCs and plant phenolics have been evaluated on different *Brassica* species, such as *Lepidium sativum*, *Eruca sativa*, *B. olerecea* var. *italica* cv. Marathon, *B. olerecea* var. *capitata* cv. coracao de boi, *B. oleracea* var. *tronchuda* cv. Tronchuda Portuguesa. Positive associations were established among specific GSL components, total GSL content and disease severity in *B. rapa* against *Xcc* [11]. This result helps to elucidate the constitutive resistance in plants against *Xcc* pathogen [17]. Here, we investigated the interaction between GSL profiles of black rot susceptible and resistant cabbage inbred lines, expression profiling of GSL biosynthesis and break-down related genes after infection by *Xcc* race 4 pathogen. These findings will be helpful for further research to elucidate the underlying mechanism of host-pathogen interactions at biochemical and molecular levels for developing black rot resistance cabbage cultivars.

## 2. Materials and Methods

### 2.1. Selection of Plant Materials 

Two cabbage genotypes SCNU-C-4072 and SCNU-C-3383 were used as source of black rot disease resistance and susceptible lines, respectively, which were selected after screening of 59 inbred cabbage lines collected from the Department of Horticulture, Sunchon National University, Korea against *Xcc* race 4 pathogen. This screening experiment was conducted in the greenhouse which had a temperature of 28 ± 2 °C, >80% humidity and 16/8 h day/night (Figure S1 and Table S1). *Xcc* race 4 (HRI-W-1279A) was collected from Horticulture Research International, Wellesbourne (HRI-W) [30]. This bacterial isolate was maintained on King’s B medium and incubated at 30 °C for 48 h [31], from which 1 × 10^8^ CFU/mL bacterial isolate was collected and used to infect 35 days old cabbage seedlings following leaf dip method after cutting 1 cm off the tip of the leaves [32]. Three biological replicates were maintained for each cabbage line and high humidity was ensured by covering the infected plants with polythene bags immediately after infection. A set of control and mock (use only water) was also maintained for each cabbage line. Disease symptoms were rated on a 0 to 3 scale after 14 days of inoculation (DAI) [30]

### 2.2. Collection of Leaf Samples for HPLC and PCR

**Figure 1:**
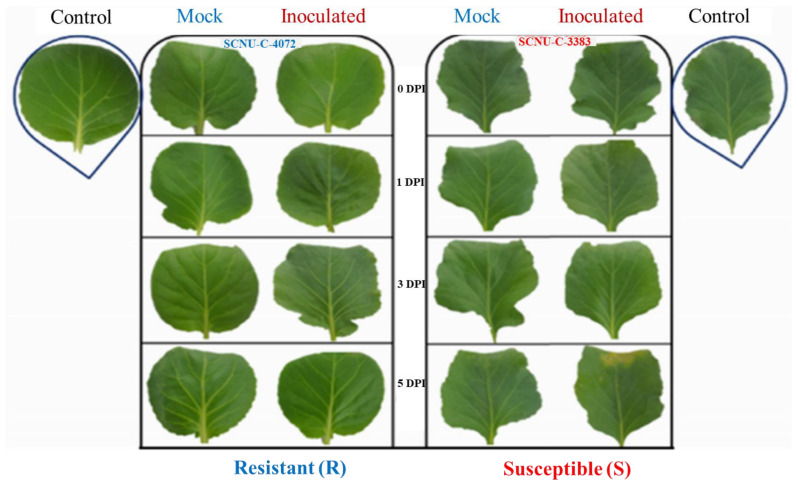
Black rot disease progress in resistant, R (SCNU-C-4072) and susceptible, S (SCNU-C-3383) cabbage lines. Thirty five (35) days old plants were inoculated with *X*. *campestris* pv. *campestris* race 4 isolate. Infected plants were observed up to 14 DAI (days after inoculation).

Leaf samples were collected from each of the control, mock-treated and *Xcc* inoculated plants, at 1, 3 and 5 DAI (Figure 1) for estimating the endogenous GSLs content and expression of GSL biosynthesis and breakdown related genes. Leaf samples were stored immediately at −80 °C after freezing in liquid nitrogen for HPLC (high performance liquid chromatography) and quantitative reverse transcription PCR (qRT-PCR) analyses. 

### 2.3. Glucosinolates Identification, Quantification and Analysis

The GSL composition of the cabbage leaf samples was estimated as desulfo-GSL from three biological replicates for each of the control, mock-treated and *Xcc* infected plants using a modified HPLC method as previously described [33,34]. Frozen leaf tissue was treated with methanol and stored at −80 °C and then ground to a very fine powder. The powdered leaf samples were stored at 70 °C for 10 min and then brought out at room temperature for 1 h. The samples were centrifuged at 10,000× *g* at 4 °C for 8 min to remove the undesirable structural components and protein molecules. The supernatant (crude GSL sample) was collected at the end of anion-exchange chromatography. Afterward, the raw GSL sample was desulfurized following the method [33,35] and the GSLs were eluted with 1 mL distilled water. The eluted desulfo-GSLs were purified by high-speed centrifugation at 20,000× *g* for 4 min at 4 °C followed by filtering through a PTFE (polytetrafluoroethylene) filter (13 mm, 0.2 μm, Advantec, Pleasanton, CA, USA). Purified GSLs were estimated by HPLC on Waters 2695 HPLC system (Waters, Milford, MA, USA) provided with a C18 column (Zorbax Eclipse XBD C18, 4.6 m ×150 mm; Agilent Technologies, Palo Alto, CA, USA). Water and acetonitrile were used as mobile phase solvents. HPLC-mass spectrometry analysis (Agilent 1200 series, Agilent Technologies) was used for identifying individual GSLs [35]. The individual GSL compound was measured at a wavelength of 229 nm using PDA 996 UV-visible detector. A standard curve was prepared with commercial standard sinigrin (SIN) for quantification of the identified individual GSLs. 

### 2.4. Expression Analysis of GSL Biosynthesis Pathway and Break-Down Related Genes

**Figure 2:**
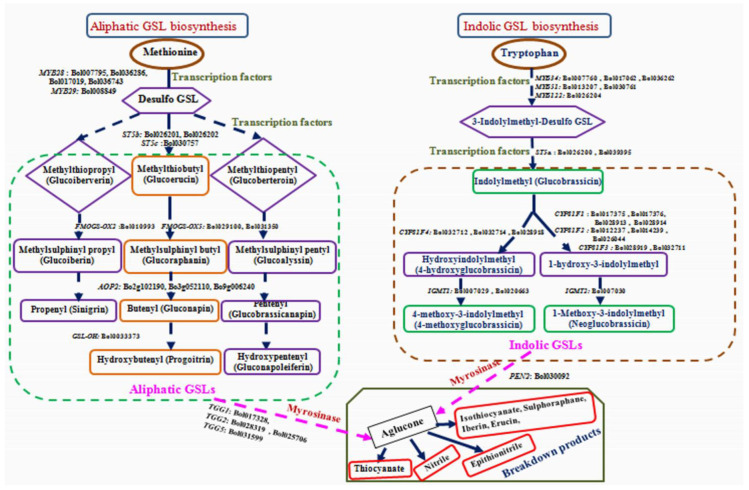
The glucosinolate pathway showing the transcription factors, glucosinolate (GSL) biosynthesis and breakdown related genes indicated aliphatic and indolic GSL biosynthesis pathways adopted from previous studies [33,35,37]. A total of 43 genes were selected (15 and 23 genes from aliphatic and indolic GSL biosynthesis pathway and 5 genes from GSL breakdown, respectively). Blue color indicates the genes, which were highly expressed in the resistant line and red color indicates the genes which were highly expressed in susceptible cabbage line after infection with *X. campestris* pv. *campestris* race 4. Bold green color number in bracket indicates 1 and 3 days after inoculation.

A total of 43 genes, of which 38 associated with GSL biosynthesis and 5 GSL breakdown were used for expression analysis. Among them, 11 genes belong to transcription factor (TF) (5 from aliphatic and 6 from indolic groups of GSL biosynthesis pathway). Primers were designed on twenty seven genes, of which 10 were from aliphatic and 17 from indolic GSL biosynthesis pathway (Figure 2 and Table S2). Primer efficiencies were assessed following the process [33,36] and highly efficient primers were used for expression analysis. 

### 2.5. Total RNA Extraction, cDNA Synthesis and Quantitative Real-Time PCR Analysis

The collected leaf samples were ground in liquid nitrogen and total RNA was extracted from 100 mg leaf tissue using RNeasy mini kit (Qiagen, Valencia, CA, USA). The quantity and quality of RNA were determined using a NanoDrop ND-1000 (260/280 nm) spectrophotometer (NanoDrop, Wilmington, DE, USA) (Supplementary Figure S2) and 1% agarose gel electrophoresis, respectively, prior to use in cDNA (complementary DNA) synthesis for qRT-PCR (Supplementary Figure S3). The cDNA was synthesized using a first-strand cDNA synthesis kit (Thermo Fisher Scientific, MA, USA) following the manufacturer’s protocol. The equality of cDNA for each sample was standardized by checking the concentration (60 ng/μL) in NanoDrop as well as comparing the band thickness of end-point PCR product of each sample amplified with *actin* genes of *B. oleracea*. Primers were designed on three actin genes named as *actin1*, *actin2*, and *actin3* with NCBI accessions AF044573 [38], JQ435879 [39] and XM_013753106 [40]. These genes were also used as internal controls for estimating relative gene expression. The qPCR was performed using a 20 μL reaction mixture contained 10 μL 2× Quanti Speed SYBR mix (Thermo Fisher Scientific), 1 μL (10 pmol) each of the forward and reverse gene-specific primers (Supplementary Table S2), 1 μL template cDNA (60 ng) and 7 μL distilled-deionized water. The PCR condition was fixed as initial denaturation at 95 °C for 10 min, 40 cycles of amplification with denaturation at 95 °C for 20 s, annealing at 58 °C for 20 s, and amplification and signal acquisition at 72 °C for 30 s. Each reaction was performed three times as technical replicates using LightCycler96 (Roche, Mannheim, Germany). Livak’s comparative 2^-ΔΔCt^ method [41] was used to calculate the relative expression level of each sample. Average Cq value of three actin genes was used for estimating relative expression of GSL biosynthesis and breakdown related genes.

### 2.6. Statistical Analysis

One-way ANOVA (analysis of variance) and mean separation following Tukey’s pairwise comparison for relative expression of each gene and GSL content at different time courses were done using Minitab 18 statistical software (Minitab Inc., State College, PA, USA). Test statistics, degrees of freedom, F- and P-values of statistical significance for GSL content and the relative expression of GSL biosynthetic and breakdown related genes are presented in Tables S3 and S4. A heat map was constructed in Microsoft Excel using conditional formatting options to display the correlation between GSL content and expression values of genes (Tables S5–S9). Principal component analysis (PCA) was done using GSL compounds as determined by HPLC and gene expression values by Minitab 18.

## 3. Results

### 3.1. Phenotypic Evaluation of Cabbage Lines Against Black Rot Disease

Fifty nine inbred cabbage lines were screened against black rot disease after infection of *Xcc* race 4. Most of the lines found as susceptible, whereas lines SCNU-C-060, SCNU-C-064 and SCNU-C-4072 were identified as resistance (Table S1). The resistant lines did not show any disease symptoms on the inoculated leaves up to 14 DAI (days after inoculation); whereas, susceptible lines produced necrotic lesions with characteristic V-shaped symptoms. One resistant (SCNU-C-4072) and one susceptible (SCNU-C-3383) line was used for further study (Figure 1 and Supplementary Figure S1).

### 3.2. Distribution of the GSL Compounds in the Black Rot Resistant and Susceptible Lines 

**Figure 3:**
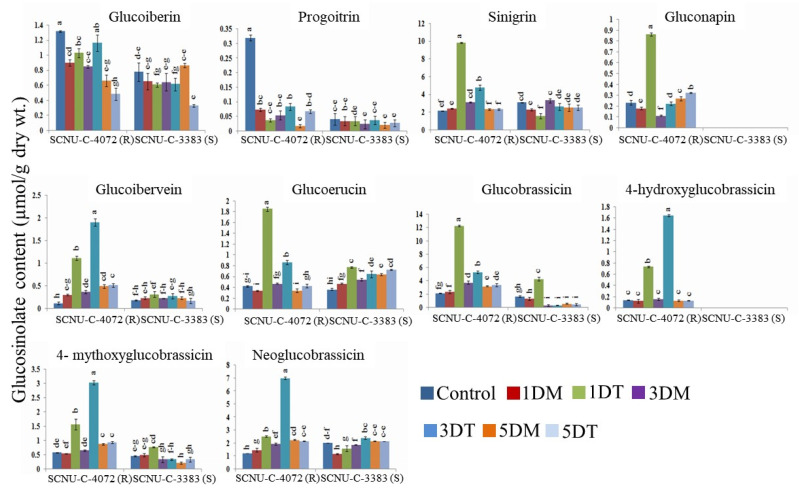
Glucosinolate contents of cabbage leaf samples from black rot resistant (SCNU-C-4072) and susceptible (SCNU-C-3383) lines under different treatments (C, control; 1DM, 1 day mock; 1DT, 1 day treated; 3DM, 3 day mock; 3DT, 3 day treated; 5DM, 5 day mock; 5DT, 5 day treated). The mean of three biological replicates is shown in the figure. Vertical bars indicate standard deviation. Different letters indicate statistically significant differences (*p* < 0.01) between genotypes (R and S lines) and treatment interactions following Tukey test. HPLC-mass spectrometry (HPLC-MS) analysis (using an Agilent 1200 series instrument, Agilent Technologies) was conducted following a previous study [34]. R, resistant; S, susceptible.

HPLC (high-performance liquid chromatography) was performed for identifying different GSL compounds in the black rot resistant (SCNU-C-4072) and susceptible (SCNU-C-3383) lines (Figure 3, Supplementary Data S1 and S2). Resistant control plants had higher amount of glucoiberin and progoitrin than susceptible line. Ten GSL compounds (glucoiberin, progoitrin, sinigrin, glucoerucin, gluconapin, glucoiberverin, hydroxyglucobrassicin, glucobrassicin, mythoxyglucobrassicin and neoglucobrassicin) were identified in the resistant line. Whereas 8 GSL compounds were identified in susceptible, except the gluconapin and hydroxyglucobrassicin lines (Figure 3). Compared to control plants, inoculated resistant and susceptible lines showed significant difference for both aliphatic and indolic GSL content. Aliphatic and indolic GSL compounds were increased significantly in the resistant line in response to *Xcc*, whereas it was decreased or not induced in the susceptible line. In the resistant line, levels of aliphatic compound (sinigrin and glucoiberverin) were increased significantly by 4.08- and 5.19-fold at 3 DAI and glucoerucin was increased 5.44-fold at 1 DAI after *Xcc* infection compared to mock (Figure 3, Tables S3 and S5). In the susceptible line, aliphatic compounds (sinigrin, glucoiberverin and glucoerucin) were decreased by 0.68-, 1.22-, and 1.64-fold, respectively, in infected plants compared to mock. The gluconapin level was increased by 4.79-fold at 1 DAI in the resistant line, whereas it was totally absent in the susceptible line (Figure 3, Tables S3 and S5). The indole GSL compounds (glucobrassicin, mythoxyglucobrassicin, and neoglucobrassicin) were increased by 5.20-fold at 1 DAI as well as 4.69- and 3.61-fold at 3 DAI, respectively, whereas hydroxyglucobrassicin was significantly increased by 10.27-fold at 3 DAI in resistant line. This indolic compound was totally absent in the susceptible line. Glucobrassicin and neoglucobrassicin were decreased by 1.02- and 1.28-fold at 1 DAI and 3 DAI, respectively, in the susceptible line. Mythoxyglucobrassicin was increased by 3.28-fold at 3 DAI in the susceptible line (Figure 3, Tables S3 and S5). Both aliphatic and indolic GSL compounds were increased in the resistant line upon *Xcc* infection compared to susceptible line.

### 3.3. Relative Expression of Transcription Factor- and GSL Biosynthesis-Related Genes in Black Rot Resistant Line 

**Figure 4:**
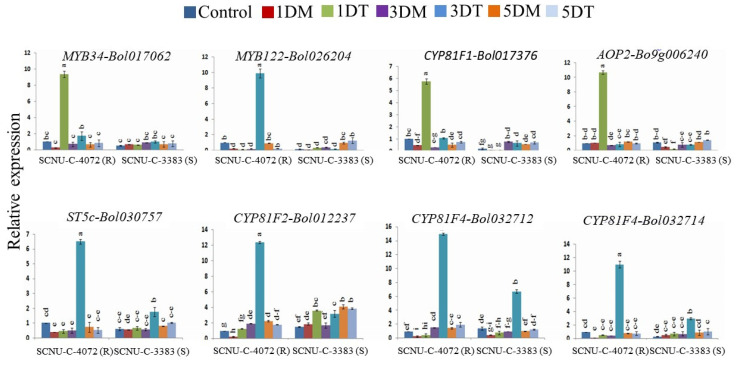
Relative expression of transcription factor-related genes and GSL biosynthesis genes in the black rot resistant (SCNU-C-4072) line at different days after inoculation (1, 3, and 5 DAI) using *Xcc* pathogen under different treatment conditions (C, control; 1DM, 1 day mock; 1DT, 1 day treated; 3DM, 3 day mock; 3DT, 3 day treated; 5DM, 5 day mock; 5DT, 5 day treated) compared to mock-treated leaves. The data were normalized using three actins (*actin 1*, *actin 2* and *actin 3*). Vertical bars indicate the standard deviation. Expression analysis was performed on three biological repeats as three technical replicates. Different letters indicate statistically significant differences (*p* < 0.01) between genotypes (R: SCNU-C-4072 and S: SCNU-C-3383) and treatment interactions following Tukey test. R, resistant; S, susceptible.

We investigated the expression patterns of selected TF (transcription factor)-related genes of the GSL biosynthesis pathway in the resistant line (SCNU-C-4072) by qPCR. Among them, eight genes showed up-regulation upon *Xcc* infection at different DAI (Figure 4 and Figure S4). Two genes (*MYB122-Bol026204* and *MYB34-Bol017062*) showed higher relative expression by 49.40- and 36.85-fold up-regulation at 1 DAI and 3 DAI, respectively, compared to mock (Figure 4 and Table S4). Two genes from aliphatic pathway; *AOP2-Bo9g006240* and *ST5c-Bol030757* showed higher expression by 10.30- and 13.12-fold up-regulation at 1 DAI and 3 DAI, respectively, compared to mock (Figure 4 and Table S7). One indolic biosynthesis gene (*CYP81F1-Bol017376*) out of four, showed significantly higher expression at 1 DAI by 11.86-fold changed, other three genes, *CYP81F2-Bol012237*, *CYP81F4-Bol032712* and *CYP81F4-Bol032714* showed higher expression by 6.40-, 9.69-, and 25.29-fold increased, respectively, at 3 DAI compared to susceptible line and mock (Figure 4 and Table S8).

### 3.4. Relative Expression of TF-Related and GSL Biosynthesis Genes in Black Rot Susceptible Line 

**Figure 5:**
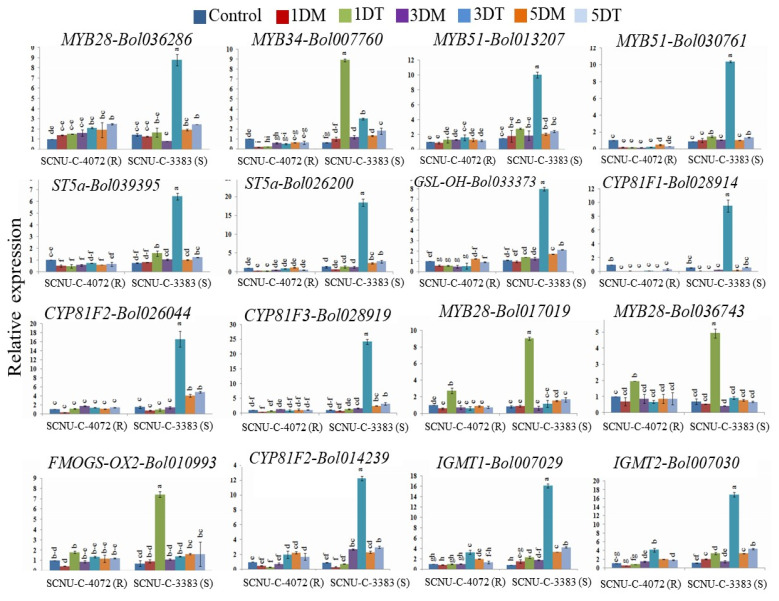
Relative expression of transcription factor-related and GSL biosynthesis genes in the black rot susceptible (SCNU-C-3383) line at different days after inoculation (1, 3, and 5 DAI) using *Xcc* pathogen under different treatment conditions (C, control; 1DM, 1 day mock; 1DT, 1 day treated; 3DM, 3 day mock; 3DT, 3 day treated; 5DM, 5 day mock; 5DT, 5 day treated) compare to mock-treated samples. Vertical bars indicate the standard deviation. Different letters indicate statistically significant differences (*p* < 0.01) between genotypes (R: SCNU-C-4072 and S: SCNU-C-3383) lines and treatment interactions following Tukey test. R, resistant; S, susceptible.

Expression level of 38 GSL biosynthetic genes was measured in the control, mock and infected cabbage plants of the susceptible line (SCNU-C-3383). Three TF-related genes, two from aliphatic GSL biosynthesis (*MYB28-Bol017019* and *MYB28-Bol036743*) and one from indolic biosynthesis (*MYB34-Bol007760*) were up-regulated by 10.38-, 8.94-, and 8.99-fold, respectively, at 1 DAI compared to mock. In addition, *MYB28-Bol036286, MYB51-Bol013207* and *MYB51-Bol030761* genes showed higher expression by 11.18-, 5.41- and 9.61-fold changed, respectively, at 3 DAI compared to mock (Figure 5, Figure S2 and Table S6). Two aliphatic GSL biosynthetic genes (*FMOGS-OX2-Bol010993* and *GSL-OH-Bol033373*) showed up-regulation upon *Xcc* inoculation compared to both control and mock samples by 7.97- and 6.29-fold changed at 1 and 3 DAI, respectively. Eight indolic GSL biosynthetic genes (*ST5a-Bol039395, ST5a-Bol026200, CYP81F1-Bol028914, CYP81F2-Bol014239, CYP81F2-Bol026044, CYP81F3-Bol028919, IGMT1-Bol007029* and *IGMT2-Bol007030*) showed higher expression in the susceptible line at 3 DAI compared to mock by 6.22-, 15.03-, 38.20-, 4.53-, 12.04-, 14.41-, 8.88- and 11.43-fold, respectively (Figure 5 and Table S8). The remaining 14 genes (*MYB28-Bol007795*, *MYB29-Bol008849*, *MYB34-Bol036262*, *ST5b-Bol026202*, *ST5b-Bol026201*, *FMOGS-OX5-Bol029100*, *FMOGS-OX5-Bol031350*, *CYP81F1-Bol028913*, *CYP81F1-Bol017375*, *CYP81F3-Bol032711*, *CYP81F4-Bol028918*, *IGMT1-Bol020663*, *AOP2-Bo2g102190*, and *AOP2-Bo3g052110*) were not induced in consistent response upon *Xcc* infection (Figure S4 and Table S6–S8).

### 3.5. Relative Expression of GSL Breakdown Related Genes in Cabbage Lines 

**Figure 6:**
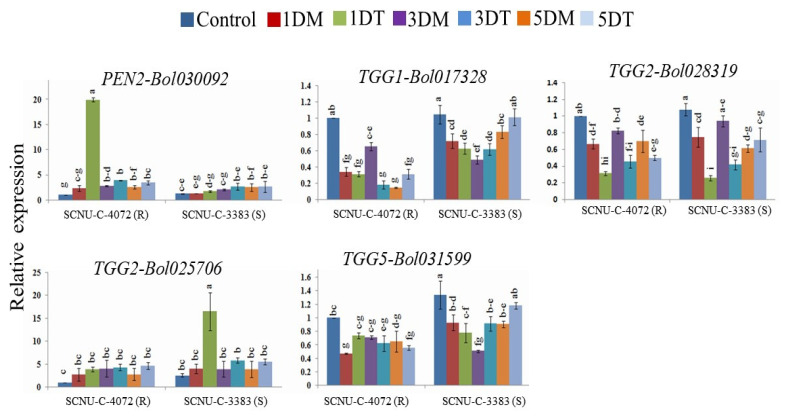
Relative expression of GSL breakdown related genes induced by myrosinase enzyme in the black rot resistant (SCNU-C-4072) and susceptible (SCNU-C-3383) cabbage lines at different days after inoculation (1, 3, and 5 DAI) using *Xcc* pathogen under different treatment conditions (C, control; 1DM, 1 day mock; 1DT, 1 day treated; 3DM, 3 day mock; 3DT, 3 day treated; 5DM, 5 day mock; 5DT, 5 day treated) compare to mock-treated leaves. Vertical bars indicate the standard deviation. Different letters indicate statistically significant differences (*p* < 0.01) between genotypes (R: SCNU-C-4072 and S: SCNU-C-3383) lines and treatment interactions following Tukey test. R, resistant; S, susceptible.

The expression of five GSL breakdown related genes showed significant variations for resistant and susceptible lines at different time courses (Figure 6 and Table S9). Gene *PEN2-Bol030092* showed significantly higher expression by 8.47-fold up-regulation at 1 DAI in the resistant line after *Xcc* infection compared to mock (Figure 6 and Table S9). Whereas, *TGG2-Bol025706* gene showed higher expression by 4.14-fold in the susceptible line at 1 DAI compared to mock. The rest of the genes; *TGG1-Bol017328, TGG2-Bol028319* and *TGG5-Bol031599,* did not show any consistent expression in resistant and susceptible lines (Figure 6).

### 3.6. Correlation between GSL Components and Expression Level of Genes in the Cabbage Lines

**Figure 7:**
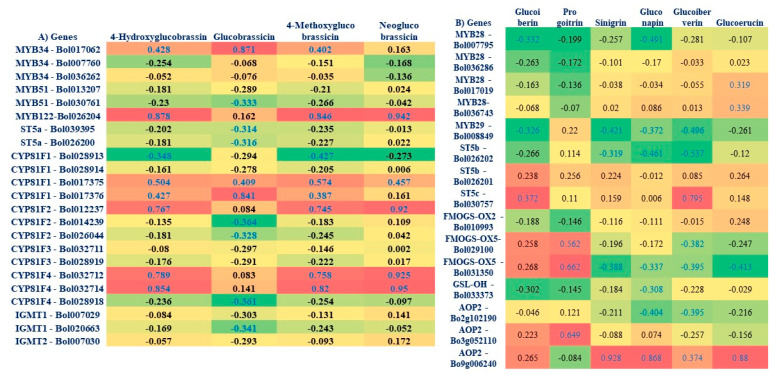
A simple heat map showing correlation between the levels of aliphatic (**A**) and indolic (**B**) glucosinolate components and expression of biosynthesis genes under different treatments (C, control; 1DM, 1 day mock; 1DT, 1 day treated; 3DM, 3 day mock; 3DT, 3 day treated; 5DM, 5 day mock; 5DT, 5 day treated) in the black rot resistant (SCNU-C-4072) and susceptible (SCNU-C-3383) cabbage lines compared to mock-treated samples. Blue colored values represent statistically significant correlations (*p* < 0.05) for each gene and individual glucosinolate combination, the values indicate the Pearson’s correlation coefficient. Red cells represent positive correlation and green cells represent negative correlation. Yellow cells represent no significant correlation. R, resistant; S, susceptible.

A simple heat map was constructed using expression of TF-related and GSL biosynthesis and breakdown genes and level of individual GSL components after *Xcc* infection in resistant and susceptible lines (Figure 7A,B). Pearson’s correlation coefficient showed significant positive associations between aliphatic GSL (glucoiberverin and glucoiberin) content and the expression level of *ST5c-Bol030757* gene (Figure 7A). Aliphatic GSL compounds (glucoiberverin, sinigrin, gluconapin and glucoerucin) and the expression of *AOP2-Bo9g006240* also showed positive association (Figure 7A and Supplementary Data S2). Similarly, aliphatic compound progoitrin and GSL biosynthesis related genes (*FMOGS-OX5-Bol029100*, *FMOGS-OX5-Bol031350* and *AOP2-Bo3g052110*) showed inconsistent positive association. In addition, a highly significant positive correlation was observed between *MYB34-Bol017062* and indolic GSL compounds (hydroxyglucobrassicin, glucobrassicin and mythoxyglucobrassicin). Significant positive associations were found between GSL compounds (hydroxyglucobrassicin, mythoxyglucobrassicin and neoglucobrassicin) and indolic GSL genes (*MYB122-Bol026204*, *CYP81F2-Bol012237*, *CYP81F4-Bol032712* and *CYP81F4-Bol032714*) (Figure 7B and Supplementary Data S2).

**Figure 8:**
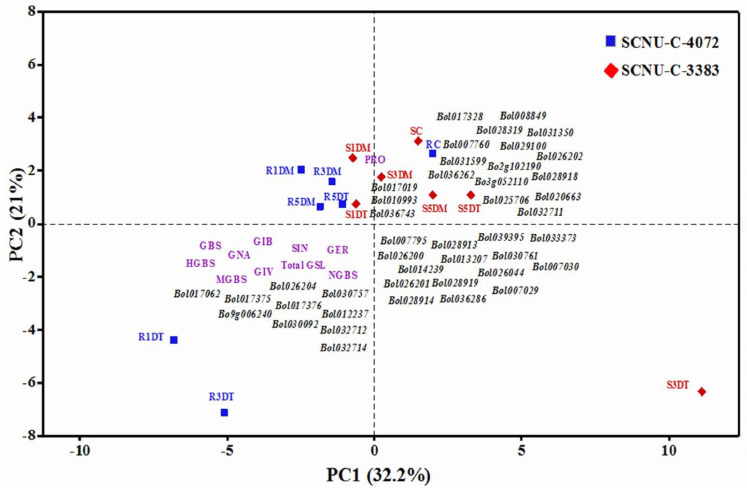
Biplot of black rot R and S cabbage lines, glucosinolate (GSL) compounds (pink color), and responses of GSL biosynthesis and breakdown related genes (black color) as determined by principle component analysis (PCA). Dark blue squares denote the mean PC scores of the R (SCNU-C-4072) line, and red diamond symbol indicate the mean PC scores of the S (SCNU-3383) line. *X. campestris* pv. *campestris* treatments denoted by C, control; 1DM, 1 day mock; 1DT, 1 day treated; 3DM, 3 day mock; 3DT, 3 day treated; 5DM, 5 day mock; 5DT, 5 day.

A principal component analysis (PCA) was done by using total GSL, GSL profiles and expression of GSL biosynthesis and breakdown related genes in two contrasting cabbage lines and identified four principal components (PCs) with an eigen value greater than unity. The first four PCs explained 79.2% of the total variance, of which 32.2%, 21.0%, 14.0% and 12.0% of the variances were included from PC1, PC2, PC3 and PC4, respectively (Table S10). The total variation found in PC1 was manifested by the higher positive coefficients of the TF, GSL biosynthesis and breakdown related genes and higher negative coefficients of GSL profiles and total GSL content in resistance and susceptible lines upon *Xcc* infection (Figure 8 and Table S10). PC1 significantly separated as resistant and susceptible groups, which was also confirmed by Tukey’s test using overall PCA score (Figure 8 and Table S10). The highly expressed GSL biosynthetic and breakdown related genes were plotted with total GSL content and most of the GSL constituents in the resistance line, as shown in the PCA biplot (Figure 8). Results indicate that resistance in cabbage might be attained by synthesizing specific GSL compounds as well as simultaneous actions of breakdown genes. 

## 4. Discussion

### 4.1. Black Rot Resistant Cabbage Lines Against Xcc

Black rot disease caused by *Xcc* is the most devastating in cabbage worldwide, causing significant crop losses [42]. Management of this disease is very difficult because of ever changing races and evolving new races of the *Xcc* pathogen. Therefore, race-specific resistance in *Brassica* crops like cabbage is important for marker-assisted breeding. We screened 59 cabbage inbred lines against race 4 of *Xcc* pathogen and the lines SCNU-C-4072 and SCNU-C-3383 were selected as resistance and susceptible, respectively (Figure S1 and Table S1).

### 4.2. GSL Compounds Varied in the Leaf Tissues of Resistant and Susceptible Cabbage Lines

We found positive association between disease resistance and GSL accumulation in contrasting cabbage lines upon infection of black rot pathogen. In both of the resistant and susceptible lines, total GSL concentration was increased significantly upon *Xcc* inoculation at different time points; 1 DAI, 3 DAI and 5 DAI (Figure 3), but amount of GSL content and components differed significantly between resistance and susceptible lines. Total GSL level was increased in leaves of *B. rapa* resistance cultivar upon infection of *Leptosphaeria maculans* and *Fusarium oxysporum*; whereas GSL was decreased in the susceptible line [43]. Recently, an increased level of GSL content was reported in resistance cabbage lines against ring spot disease [34]. A similar result was also found in the susceptible *B. napus* cultivars infected with *Alternaria brassicae* pathogen [43,44]. In a previous study, a positive association was found between pathogen-induced indolic GSL compounds and infection of *Sclerotinia sclerotiorum* [45]. In the case of plant pathogenic bacteria, myrosinase enzyme mediated GSL hydrolyzed products to act as tolerant against *Xcc* [11,17]

Aliphatic GSL (sinigrin, gluconapin, glucoiberverin and glucoerucin) and indolic (glucobrassicin, hydroxyglucobrassicin, mythoxyglucobrassicin and neoglucobrassicin) content was found to increase due to *Xcc* infection in resistant cabbage line (Figure 3) and represents its involvement in improving resistance against *Xcc* pathogen in cabbage. Aliphatic GSL (glucoiberverin, indolic; glucobrassicin and mythoxyglucobrassicin) was found to increase in cabbage lines resistant to *Mycosphaerella brassicicola* [34]. The aliphatic GSL compound, sinigrin showed a potential role in inhibiting the growth of *S. sclerotiorum* in kale and *Arabidopsis* [23,46]. It was reported that the aliphatic gluconapin can defend *B. rapa* plants against *Xcc* pathogen [17], thereby a higher amount of gluconapin and hydroxyglucobrassicin was found in resistant *B. rapa* cultivar [43]. In kale, higher hydroxyglucobrassicin exhibited resistant to the *Xcc* pathogen [46]. A study on kale and *Xcc* pathogen interactions suggested that the indolic GSLs compound (glucobrassicin) showed as more effective than aliphatic GSLs in disease resistance [23]. Therefore, it could be concluded that increased level of aliphatic and indolic compounds may confer resistance against *Xcc* pathogen in cabbage.

### 4.3. Black Rot Pathogen Induced GSL Biosynthesis Genes in Contrasting Cabbage Lines

The qPCR profiles showed differential gene expression in the black rot resistant and susceptible cabbage lines (Figure 2, Figure 4, Figure 5 and Figure 6). Eight genes showed consistent higher relative expression level in the resistant cabbage line (Figure 2 and Figure 4). Among them, two TF, 2 aliphatic GSL and four genes were involved in indolic GSL biosynthesis. Similarly, enhanced expression was found in *MYB34-Bol017062*, *MYB122-Bol026204* and *ST5c-Bol030757* genes in the resistant cabbage line, which conferred resistance to ring spot and white mold diseases, respectively [34,36].

### 4.4. Association of Aliphatic GSL Genes with Individual GSL Compound in Black Rot Resistance

Up-regulation of *ST5c-Bol030757* and *AOP2-Bo9g006240* genes led to increase aliphatic GSL content in the black rot resistant line upon *Xcc* infection. Increased expression of aliphatic GSL biosynthesis genes, *ST5b* and *ST5c* were found to be involved directly in alteration of desulfoglucosinolates to glucoiberin and glucoiberverin [47]. In this study, increased expression of *ST5c-Bol030757* was associated with higher levels of glucoiberverin content in resistant line (Figure 7A and Supplementary data S2) supported by the reports [33,36]. Moreover, *AOP2* gene converts methylsulphinylalkyl glucosinolates to sinigrin and gluconapin [47]. In *B. rapa*, overexpression of *AOP2* gene catalyzes conversion of beneficial glucoraphanin to gluconapin [37]. In *Arabidopsis*, *AOP2* gives positive feed-back in regulation of controlling glucosinolate biosynthesis by optimizing the resources in defensive metabolites [48]. *AOP2* gene expression showed positive relation between up-regulation of the gene and increased glucoerucin content in cabbage inbred lines [33]. Our results also suggested that *Xcc* induced up-regulation of genes and increased level of aliphatic compounds may have a link to black rot resistance in cabbage (Figure 7A and Supplementary data S2). However, detailed molecular studies are needed for precision of this association either by gene editing or knock-out mutation of the particular gene.

### 4.5. Association of Indolic GSL Genes and Individual GSL Compounds in Black rot Resistance

In this study, two TF (*MYB122-Bol026204* and *MYB34-Bol017062*) and four indolic GSL biosynthesis genes (*CYP81F1-Bol017376, CYP81F2-Bol012237*, *CYP81F4-Bol032712* and *CYP81F4-Bol032714*) were increased significantly in the resistant line upon *Xcc* infection, which lead to an increase in the indolic compounds (glucobrassicin, hydroxyglucobrassicin, methoxyglucobrassicin and neoglucobrassicin) (Figure 7B and Supplementary data S2). The *MYB34* gene regulates indolic GSL biosynthesis directly in *Arabidopsis* [49] and in *B. oleracea* [33,35]. *MYB34* together with *MYB51* and *MYB122* regulated resistance in *Arabidopsis* after *Plectosphaerella cucumerina* infection and the indolic GSL biosynthesis genes were triggered by GSL breakdown products induced by *PEN2* gene [50]. Accumulation of indolic GSL compound tends to up-regulate *CYP81F1*, *CYP81F2*, and *CYP81F4* genes in the resistant line (Figure 2, Figure 3 and Figure 7B), which is supported by the association between expression of *CYP81F1-Bol017376*, *CYP81F2-Bol012237* genes and glucobrassicin content in *B. oleracea* [34,50]. Methoxyglucobrassicin level was increased up to 30–47% in response to blackleg pathogen in *B. napus* after 5–8 days of inoculation [51]. Anti-fungal activity of methoxyglucobrassicin, glucobrassicin and sinigrin was reported in an in vitro analysis [52]. In this study, the indolic GSLs (glucobrassicin, methoxyglucobrassicin, hydroxyglucobrassicin and neoglucobrassicin) were found to increase in resistant line upon *Xcc* infection (Figure 7B and Supplementary data S2), suggesting their role in conferring resistance against *Xcc* pathogen in cabbage. Since, *CYP81F1-Bol017376*, *CYP81F2-Bol012237*, *CYP81F4-Bol032712* and *CYP81F4-Bol032714* genes were involved in methoxylation and conversion of glucobrassicin to 4-methoxyglucobrassicin, 4-hydroxyglucobrassicin and neoglucobrassicin, which may boost-up resistance in cabbage. Sixteen GSL biosynthesis genes were highly up-regulated in susceptible line (Figure 2 and Figure 5) and may be involved in the susceptibility to *Xcc* in cabbage. These results are consistent with low accumulation of individual GSL compound and lower expression of GSL biosynthesis genes in cabbage lines and vice-versa [33].

### 4.6. Expression of GSL Breakdown Related Genes in Cabbage Lines after Xcc Inoculation

Glucosinolate breakdown related gene (*PEN2-Bol030092*) showed significant up-regulation in black rot resistant line at 1 DAI (Figure 2 and Figure 6, and Table S7). A previous study reported that *PEN2* gene is involved in resistance in *Arabidopsis* via callose deposition and glucosinolate stimulation [53]. Moreover, accumulation of indolic GSL compound in *Arabidopsis* after fungal infection is triggered by the myrosinase *PEN2* gene [54]. *PEN2* plays a key role in triggering the expression of indolic GSL biosynthesis genes in response to necrotrophic fungal pathogen [50]. The GSL hydrolysis products induced by myrosinase were reported to act as tolerance against *Xcc* [11,17]. The GSL-myrosinase defense system could be altered by the expression of GSL biosynthesis and breakdown related genes and enhance resistance to herbivores [54]. Our PCA results also confirmed the relations of susceptibility and resistance reactions with different GLS compounds and up-regulation of the respective gene at particular time courses.

## 5. Conclusions

A positive association between expression of GSL biosynthesis and breakdown related genes and individual GSL compound was found in resistant and susceptible cabbage lines upon infection by *Xcc* race 4. Aliphatic (glucoiberverin, sinigrin, gluconapin and glucoerucin) and indolic (glucobrassicin, hydroxyglucobrassicin, methoxyglucobrassicin and neoglucobrassicin) compounds were associated positively with black rot resistance in cabbage. Individual GSL compounds and their associated genes could be putative candidates for biochemical and genetic stimuli of black rot resistance in cabbage. The predicted candidate genes could be further confirmed by functional characterization to improve black rot resistance breeding in cabbage.

## Supplementary Materials

The following are available online at https://www.mdpi.com/2223-7747/9/9/1121/s1, Figure S1: Phenotype screening of selected black rot resistant (SCNU-C-4072) and susceptible (SCNU-C-3383) cabbage lines after inoculation with *X. campestris* pv. *campestris* race 4. Photograph was taken at 14 days after inoculation (DAI). Black rot-resistant (R) and -susceptible (S) lines were defined according to the disease scoring scale (0-3), Figure S2: RNA measurement of leaf samples (Black rot) by NanoDrop (ND-1000 Spectrophotometer) (C, control; 1DM, 1 day mock; 1DT, 1 day treated; 3DM, 3 day mock; 3DT, 3 day treated; 5DM, 5 day mock; 5DT, 5 day treated). R: Resistant; S: Susceptible, Figure S3: RT-PCR amplification of black rot cDNA sample of cabbage R (SCNU-C-4072) and S (SCNU-C-3383) lines by three *actins* (*actin* 1, 2, and 3) primer of *Brassica oleracea*. (C, control; 1DM, 1 day mock; 1DT, 1 day treated; 3DM, 3 day mock; 3DT, 3 day treated; 5DM, 5 day mock; 5DT, 5 day treated). R: Resistant; S: Susceptible. M: 100 bp DNA ladder, Figure S4: Inconsistent response of expression of transcription factor related and glucosinolate biosynthesis genes in black rot R (SCNU-C-4072) and S (SCNU-C-3383) lines of cabbage. C, control; 1DM, 1 day mock; 1DT, 1 day treated; 3DM, 3 day mock; 3DT, 3 day treated; 5DM, 5 day mock; 5DT, 5 day treated. The mean of three biological replicates are used. Vertical bars indicate standard deviation. Different letters indicate statistically significant differences between R and S lines and treatment interactions. R: Resistant; S: Susceptible, Table S1: Phenotypic performance of cabbage (*Brassica oleracea* var. *capitata*) lines after inoculation with *Xcc* race 4 to identify the black rot resistance cabbage lines following by the most popular disease scoring scale based on lesion size produced on the leaves (0–3: 0 = R, 1 = MR, 2 = S and 3 = HS) (Vicente et al. 2001). Cabbage leaves rated 0 for resistant (R), 1 for moderately resistant (MR), 2 for susceptible (S) and 3 for highly susceptible (HS). Black bold color resistant and susceptible lines were selected for GSL profiling and GSL biosynthesis including with GSL break-down related gene expressions, Table S2: Primer sequences and efficiency (Abuyusuf et al. 2018; Robin et al. 2016) for the 43 glucosinolate (GSL) biosynthesis and GSL break-down related genes including three *actin* genes (*actin1*, *actin2* and *actin3*) used in the relative expression analysis through q-PCR in black rot resistant (SCNU-C-4072) and susceptible (SCNU-C-3383) lines, Table S3: Test statistic F and P-values for glucosinolate component in black rot resistant (R) line SCNU-C-4072 and susceptible (S) line SCNU-C-3383 of cabbage at a significance level of α = 0.05, respectively, Table S4: Test statistic F and P-values for expression of glucosinolate biosynthesis and myrosinase genes in black rot resistant (R) line SCNU-C-4072 and susceptible (S) line SCNU-C-3383 of cabbage at a significance level of *P* < 0.05, respectively, Table S5: Heat maps comparing black rot resistant (SCNU-C-4072) and susceptible SCNU-C-3383) lines shows fold changes in individual glucosinolate component in *X.campestris* pv. *campestris*–inoculated leaf samples compared to respective mock-treated samples.1 Day, T1/M1; 3 Day, T3/M3, 5 Day, T5/M5; M1, 1 day mock; T1, 1 day treated; M3, 3 day mock; T3, 3 day treated; M5, 5 day mock; T5, 5 day treated, Table S6: Heat maps comparing black rot resistant line SCNU-C-4072 and susceptible line SCNU-C-3383 shows fold changes in expression of transcription factor related genes in *X. campestris* pv. *campestris-inoculated* leaf samples compared to respective mock-treated samples. 1 Day, T1/M1; 3 Day, T3/M3, 5 Day, T5/M5; M1, 1 day mock; T1, 1 day treated; M3, 3 day mock; T3, 3 day treated; M5, 5 day mock; T5, 5 day treated, Table S7: Heat maps comparing black rot resistant line SCNU-C-4072 and susceptible line SCNU-C-3383 shows fold changes in expression of aliphatic glucosinolate biosynthesis genes in *X. campestris* pv. *campestris*-inoculated leaf samples compared to respective mock-treated samples. 1 Day, T1/M1; 3 Day, T3/M3, 5 Day, T5/M5; M1, 1 day mock; T1, 1 day treated; M3, 3 day mock; T3, 3day treated; M5, 5 day mock; T5, 5 day treated, Table S8: Heat maps comparing black rot resistant line SCNU-C-4072 and susceptible line SCNU-C-3383 shows fold changes in expression of indolic glucosinolate biosynthesis genes in *X. campestris* pv. *campestris*-inoculated leaf samples compared to respective mock-treated samples. 1 Day, T1/M1; 3 Day, T3/M3, 5 Day, T5/M5; M1, 1 day mock; T1, 1 day treated; M3, 3 day mock; T3, 3 day treated; M5, 5 day mock; T5, 5 day treated, Table S9: Heat maps comparing black rot resistant line SCNU-C-4072 and susceptible line SCNU-C-3383 shows fold changes in expression of glucosinolate break-down related genes in *X. campestris* pv. *campestris*-inoculated leaf samples compared to respective mock-treated samples. 1 Day, T1/M1; 3 Day, T3/M3, 5 Day, T5/M5; M1, 1 day mock; T1, 1 day treated; M3, 3 day mock; T3, 3 day treated; M5, 5 day mock; T5, 5 day treated, Table S10: Component loadings of cabbage black rot resistant (R;SCNU-C-4072) and susceptible (S;SCNU-C-3383) lines, glucosinolate compounds and glucosinolate biosynthesis and GSL break-down related gene responses as determined by the principal component analysis (PCA) Tukey test was done based on *p* < 0.05, Supplementary file 1: Mass spectrometry analysis (HPLC/MS, Agilent 1,200 series, Agilent Technologies) of leaf samples from black rot R line SCNU-C-4072 (1-7) and S line SCNU-C-3383 (8-14) of cabbage under different treatments (C, control; 1DM, 1 day mock; 1DT, 1 day treated; 3DM, 3 day mock; 3DT, 3 day treated; 5DM, 5 day mock; 5DT, 5 day treated) was used to identify individual GSLs. R: Resistant; S: Susceptible, Supplementary file 2: Correlation matrix value for GSL genes and individual GSL content.
