# Supplementary material for: Glucosinolate Profile and Glucosinolate Biosynthesis and Breakdown Gene Expression Manifested by Black Rot Disease Infection in Cabbage
Source: Plants (Basel). 2020 Aug 30;9(9):1121. doi: 10.3390/plants9091121 (PMC7569847; doi:10.3390/plants9091121)
Supplement: Supplementary file 1 [file plants-09-01121-s001.zip › Supplementary materials/Supplementary data S1.pptx]

## Slide 1
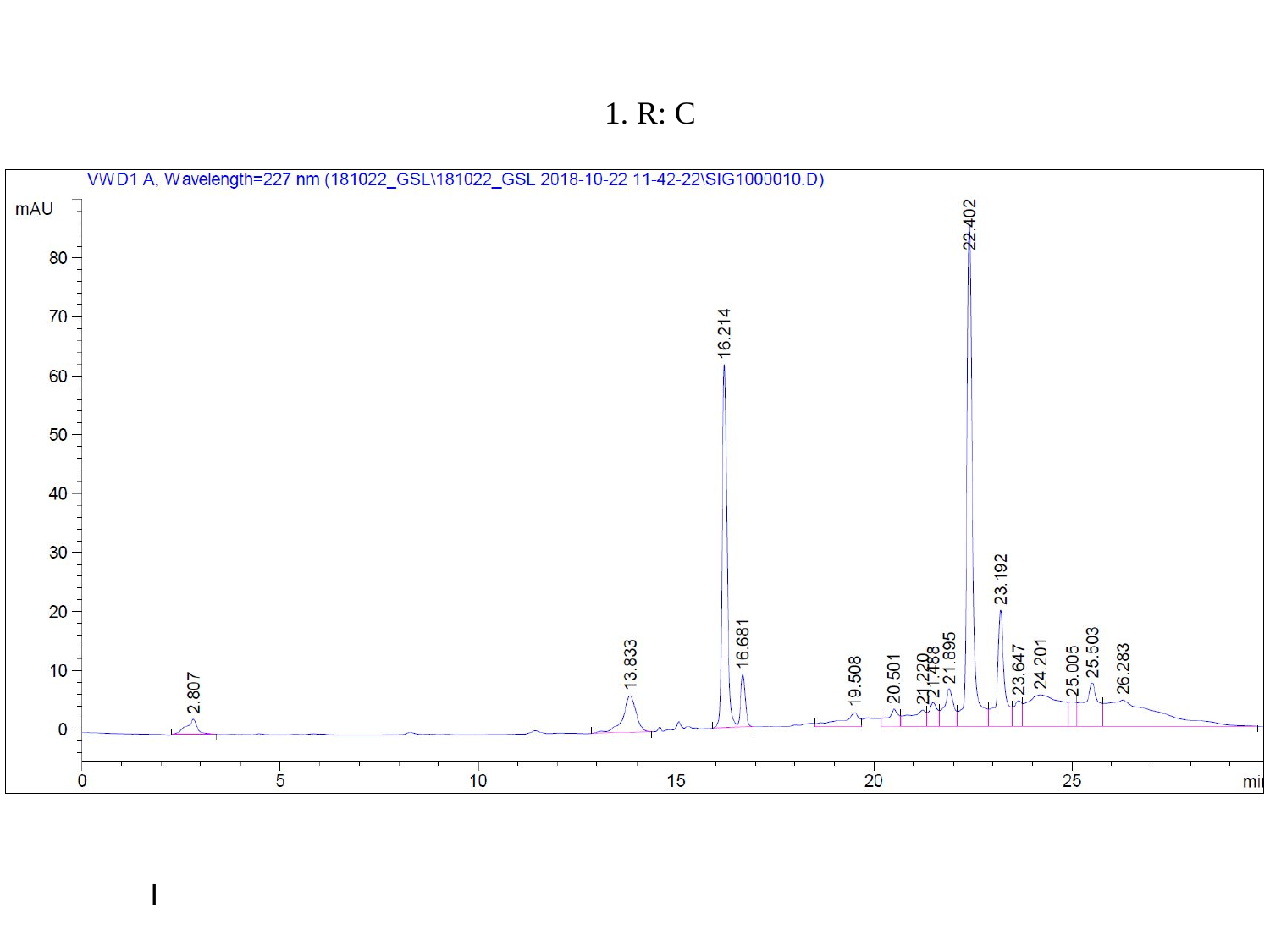

1. R: C
I

## Slide 2
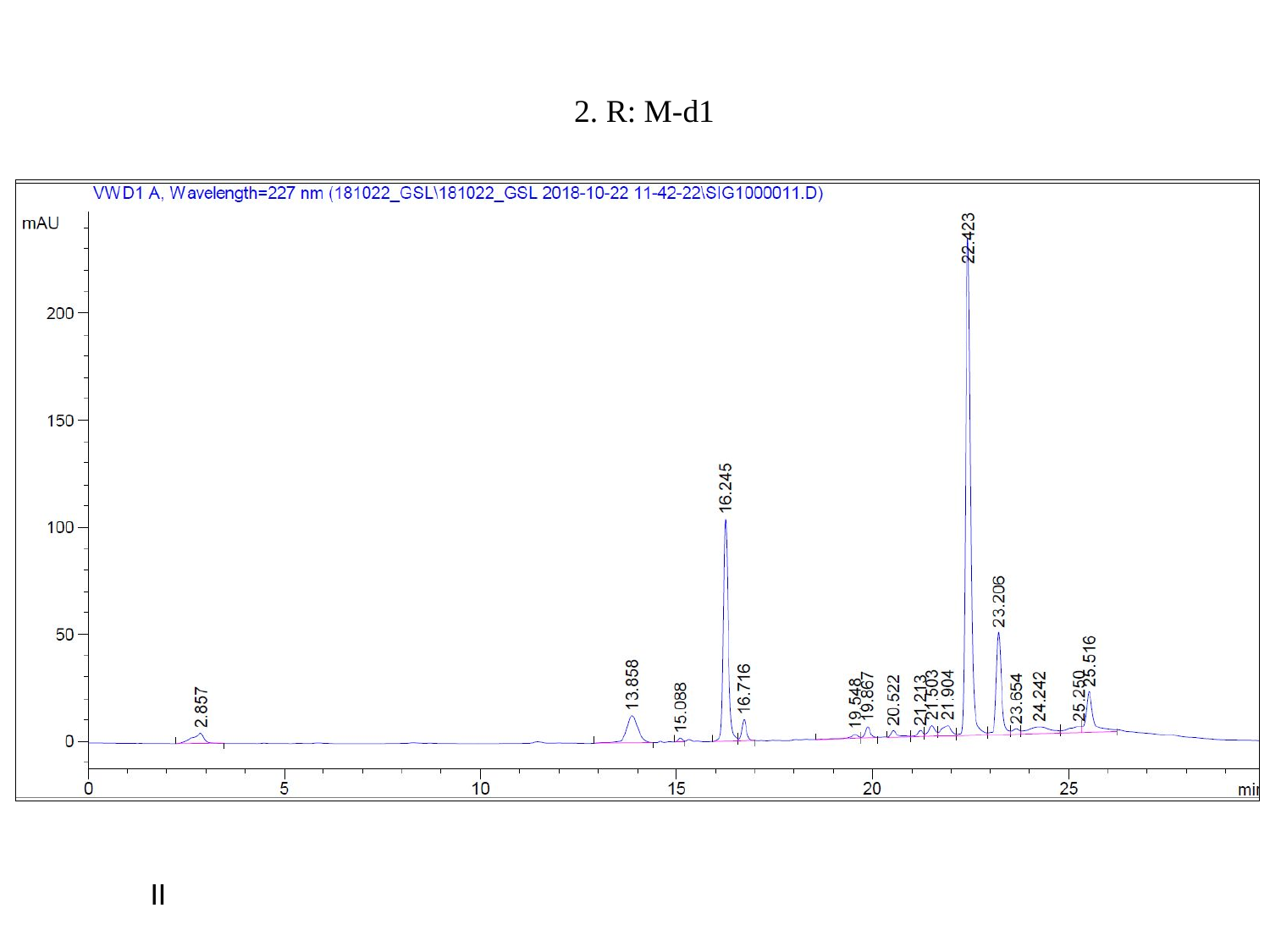

2. R: M-d1
II

## Slide 3
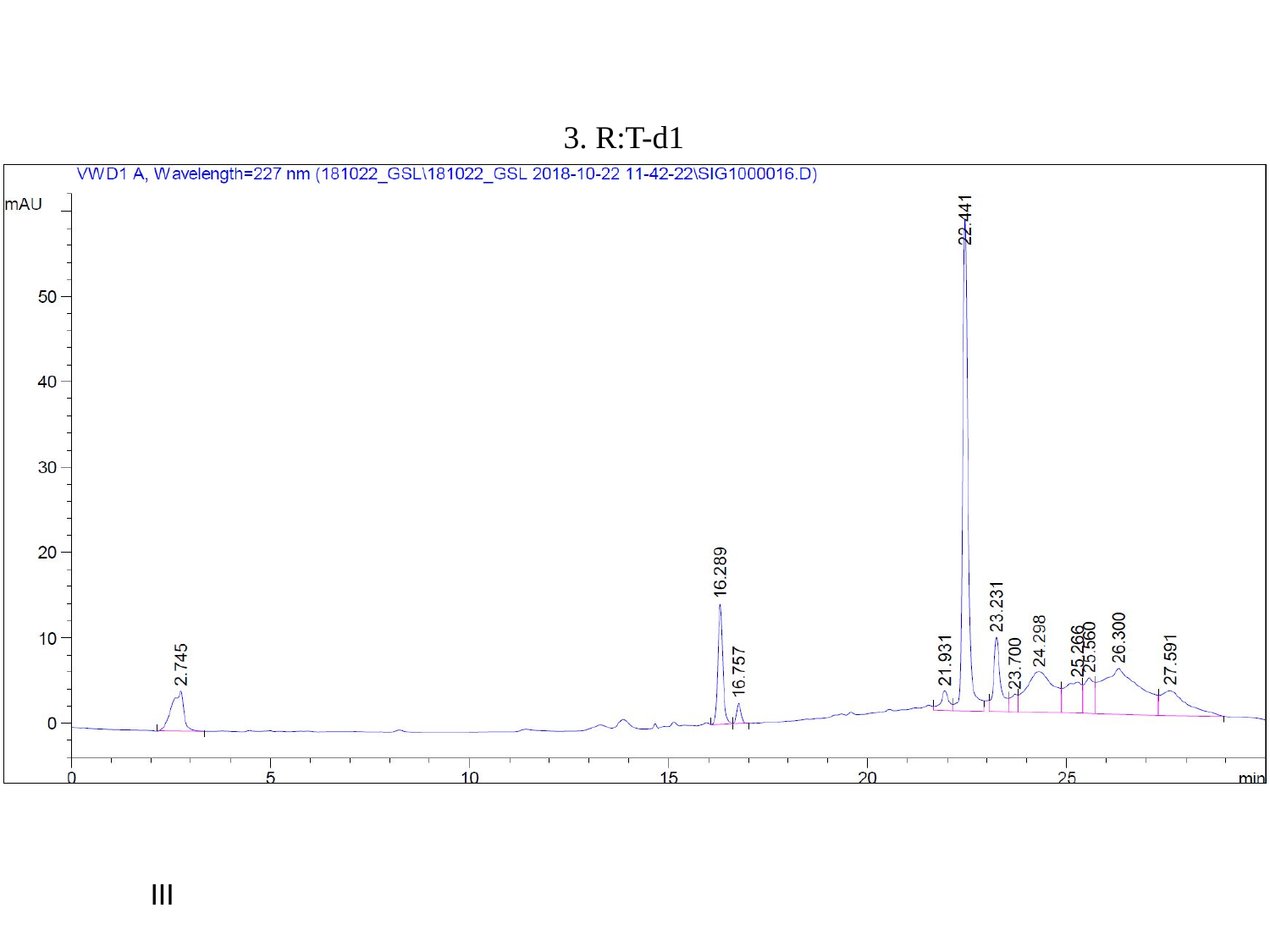

3. R:T-d1
III

## Slide 4
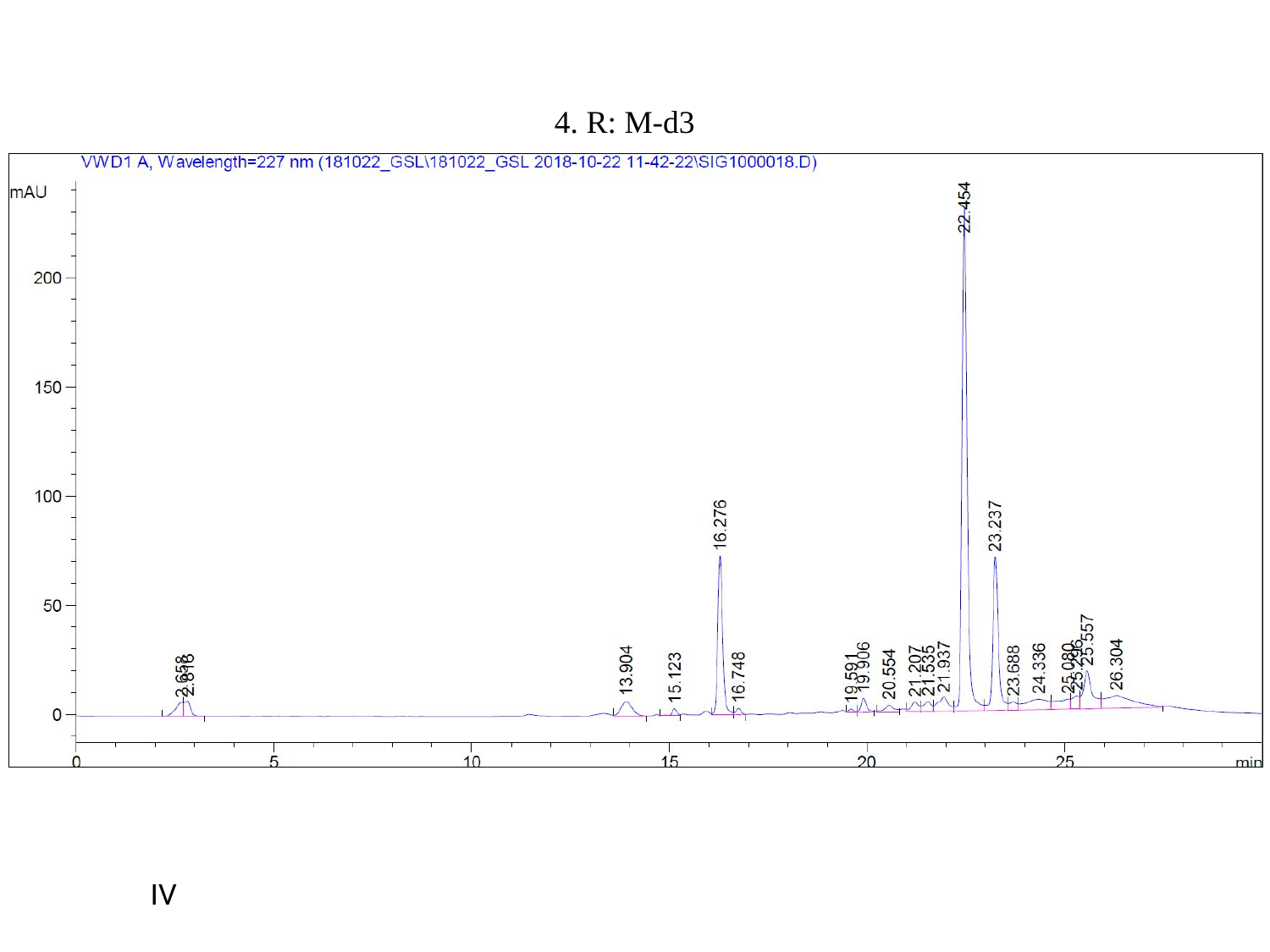

4. R: M-d3
IV

## Slide 5
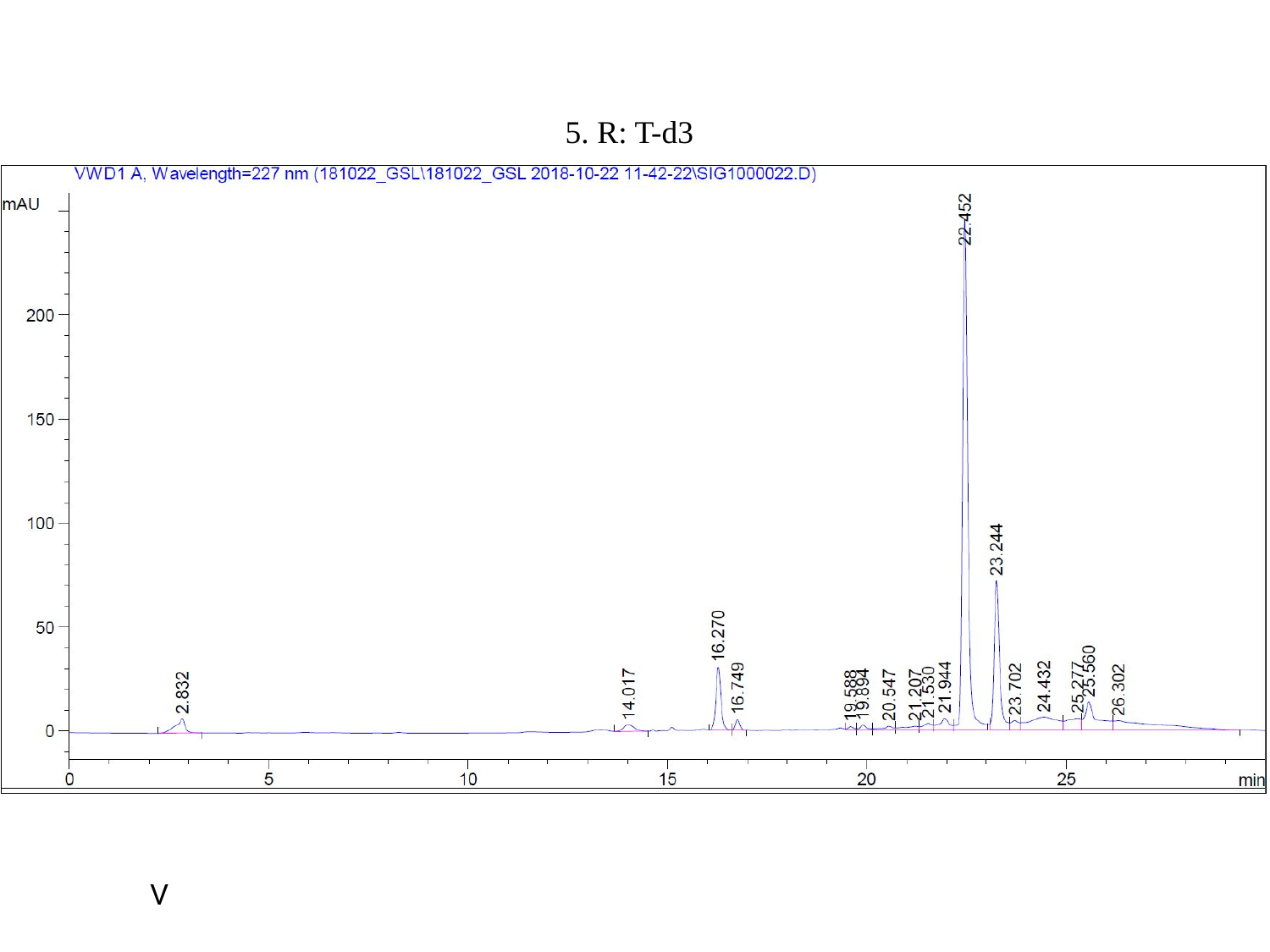

5. R: T-d3
V

## Slide 6
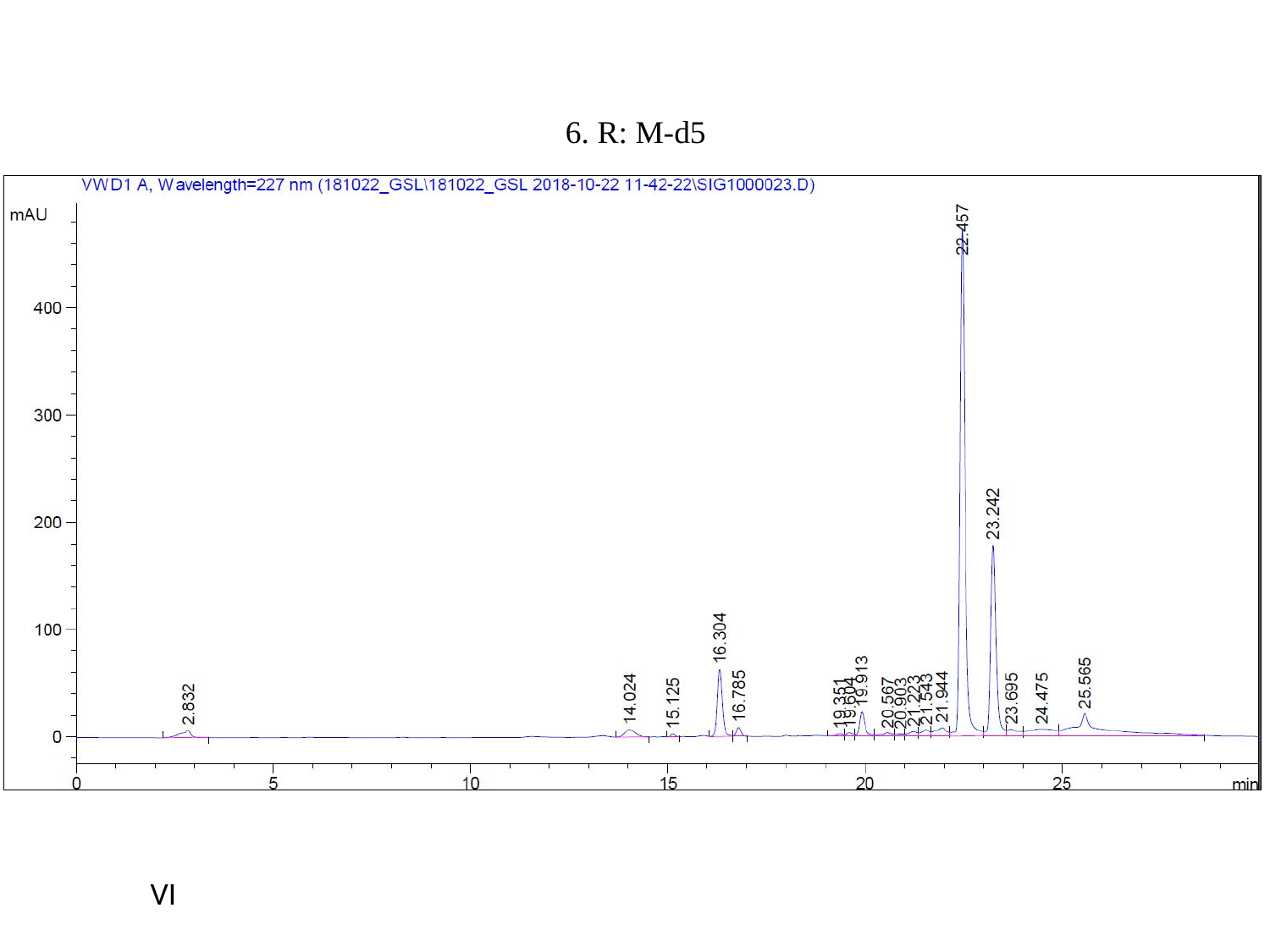

6. R: M-d5
VI

## Slide 7
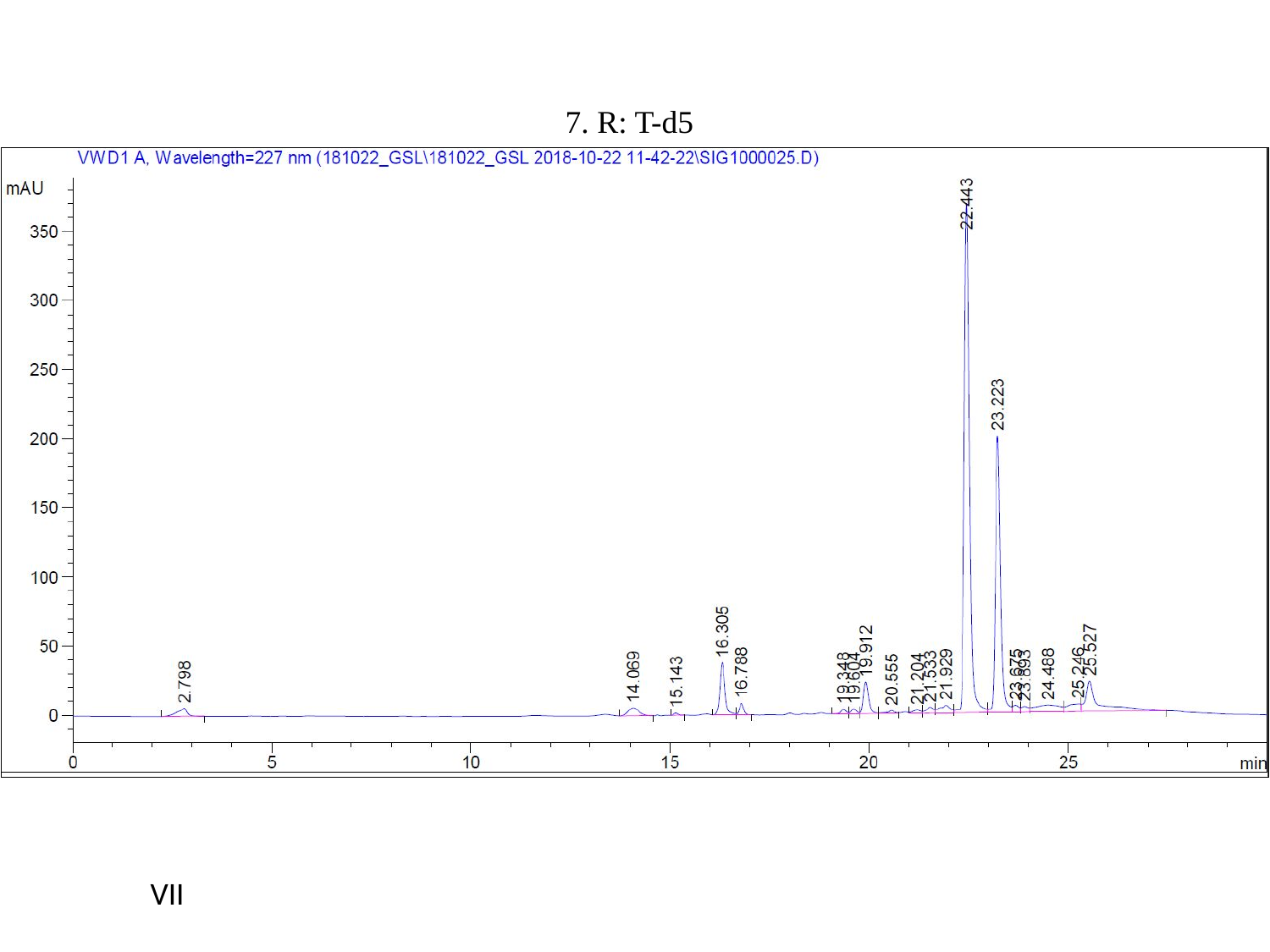

7. R: T-d5
VII

## Slide 8
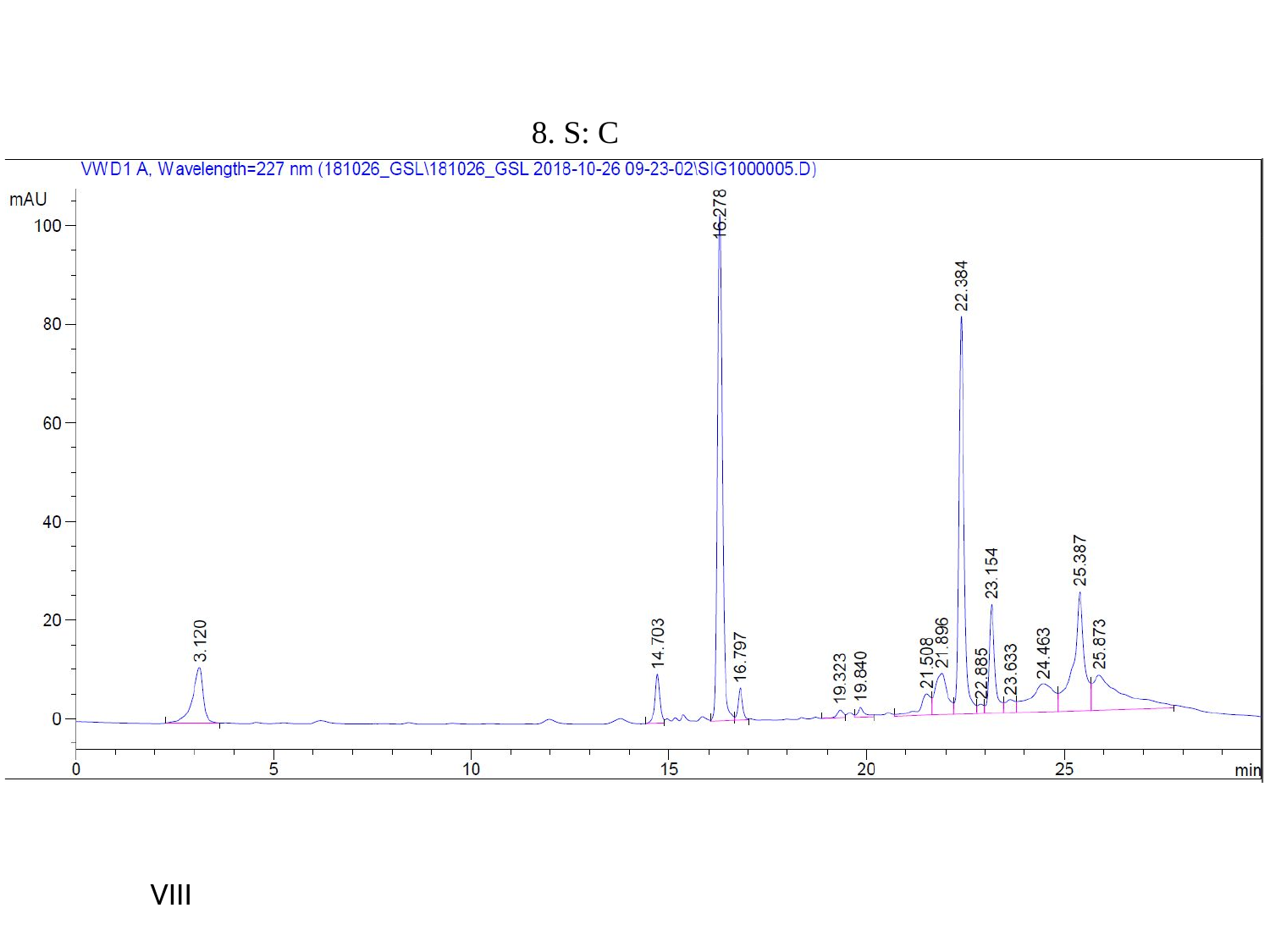

8. S: C
VIII

## Slide 9
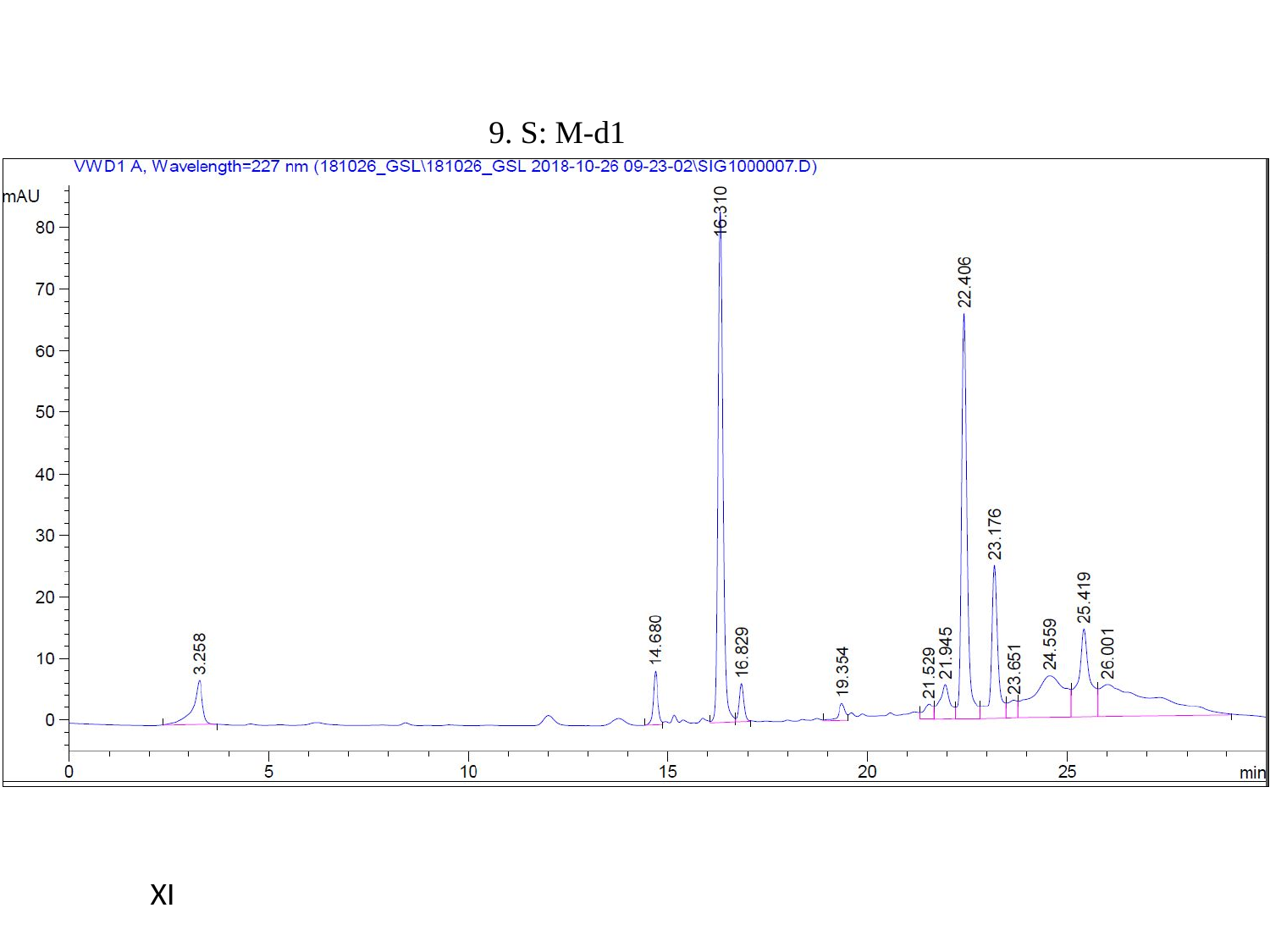

9. S: M-d1
XI

## Slide 10
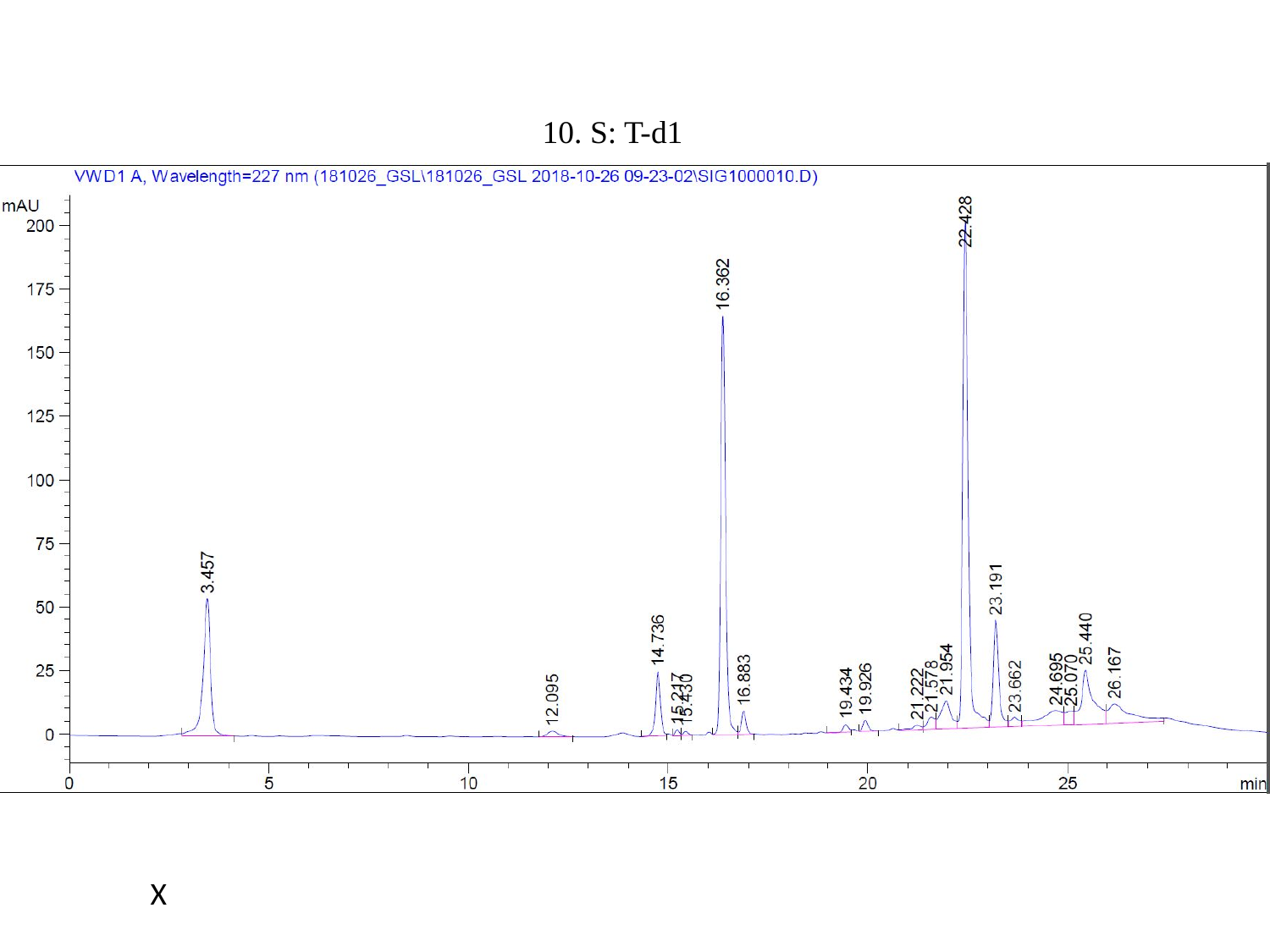

10. S: T-d1
X

## Slide 11
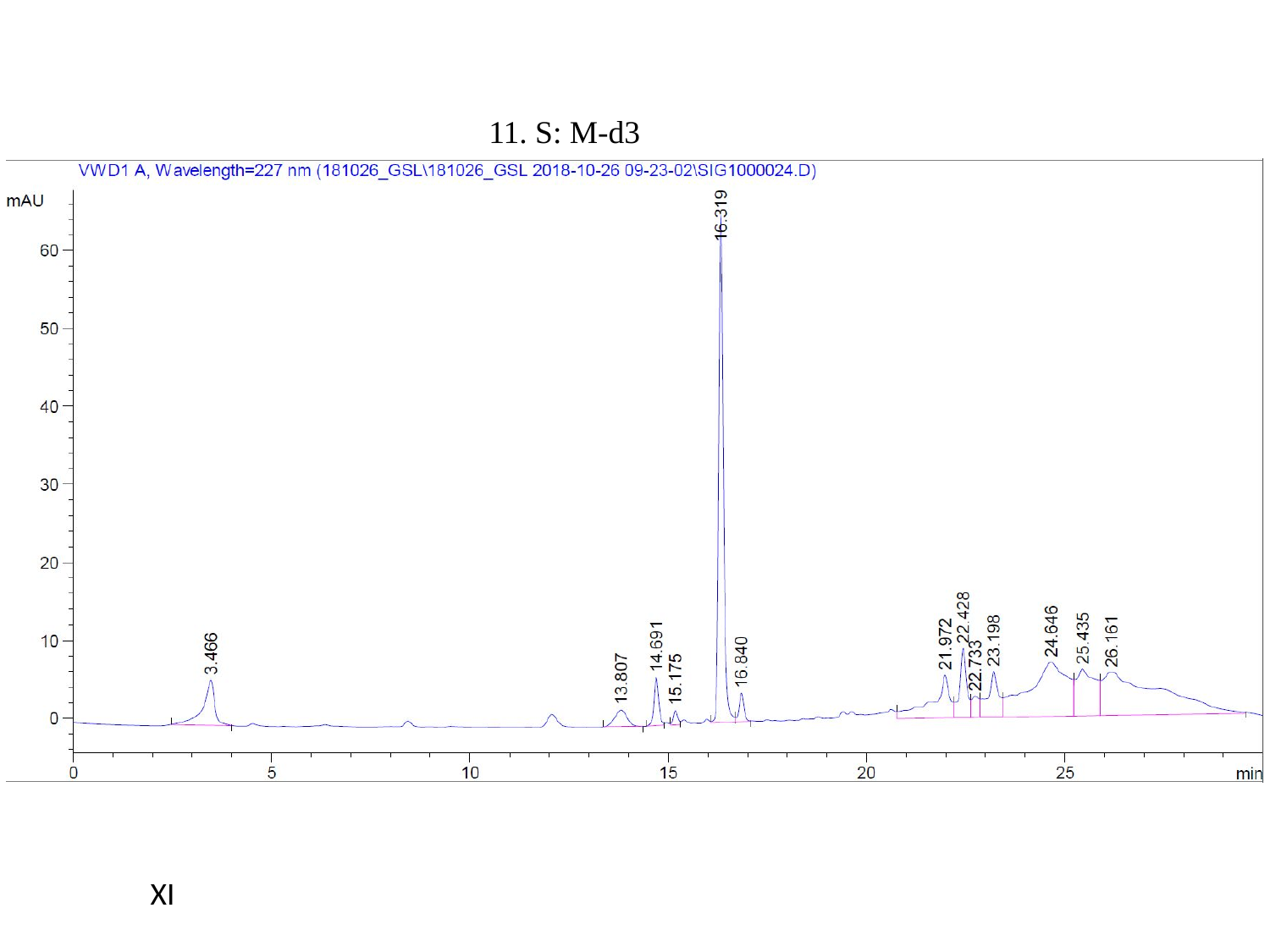

11. S: M-d3
XI

## Slide 12
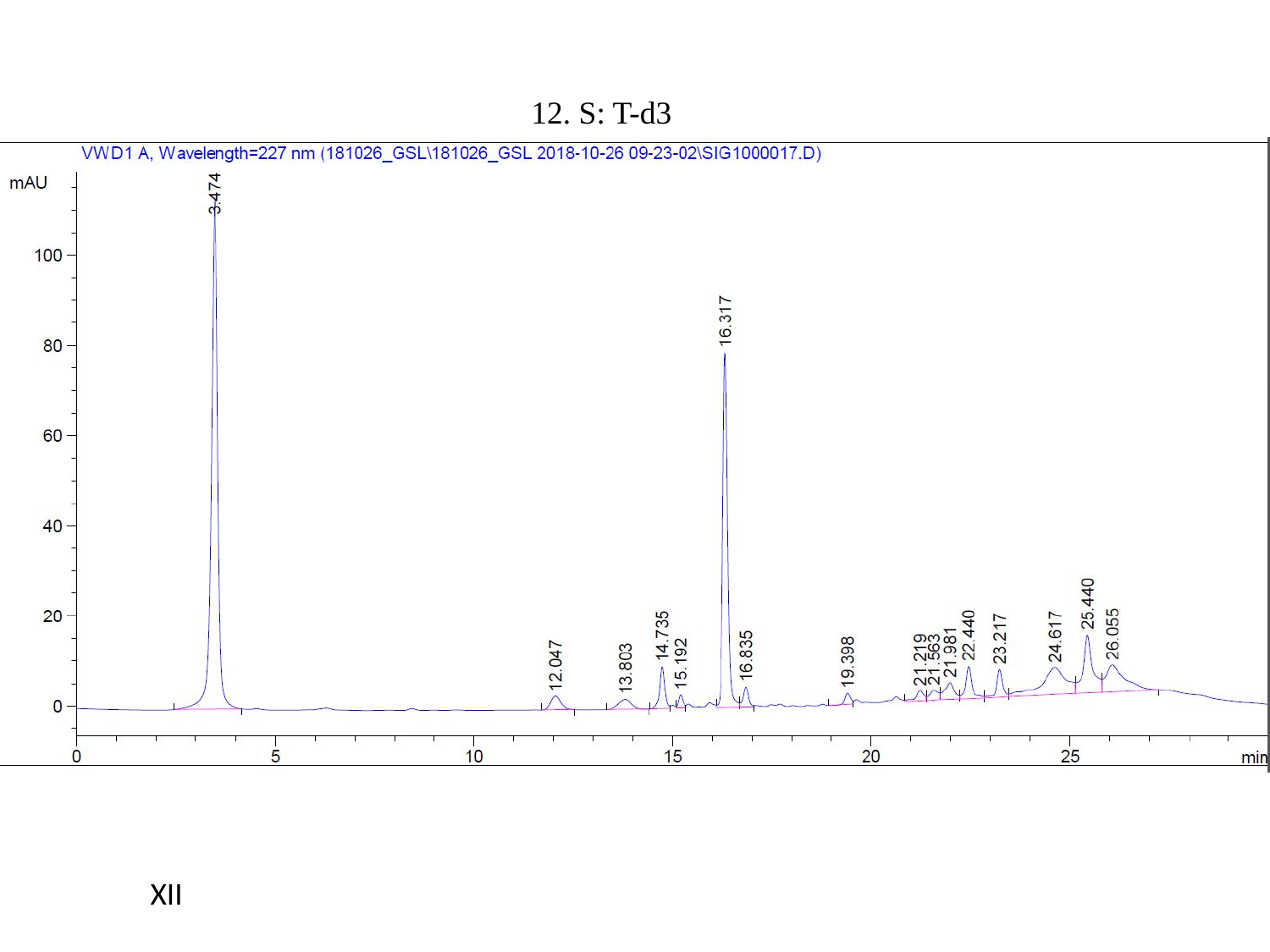

12. S: T-d3
XII

## Slide 13
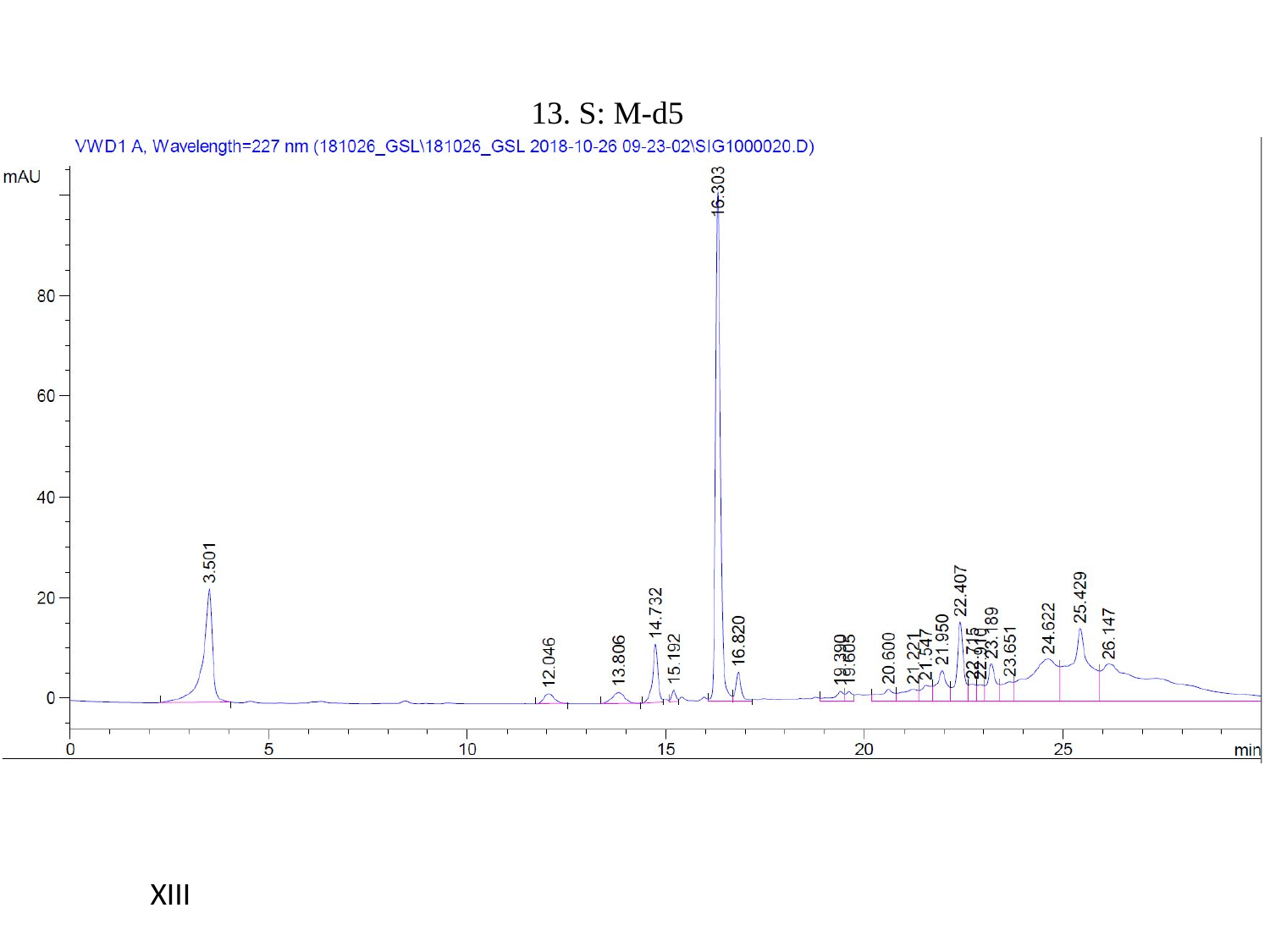

13. S: M-d5
XIII

## Slide 14
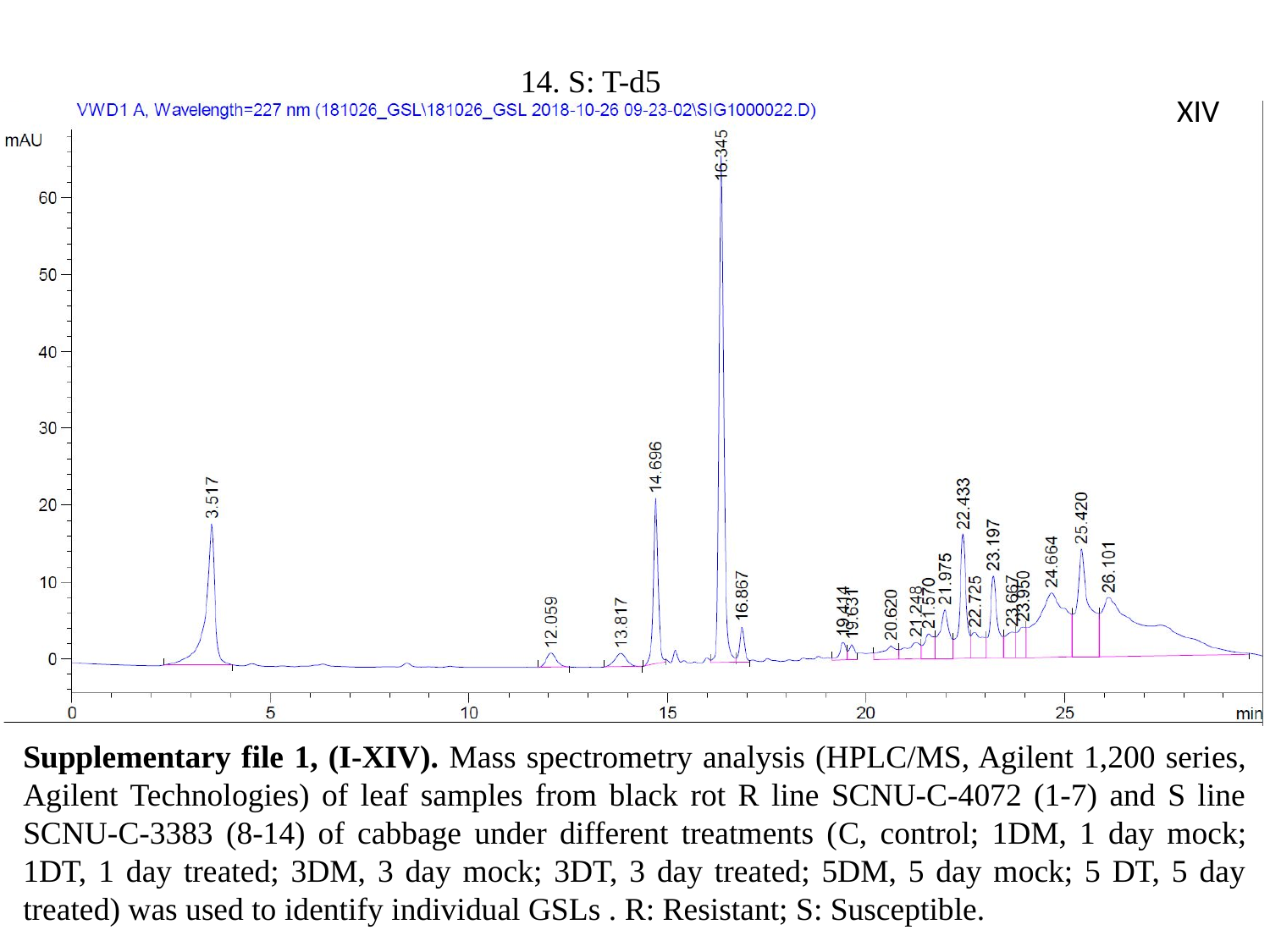

14. S: T-d5
XIV
Supplementary file 1, (I-XIV). Mass spectrometry analysis (HPLC/MS, Agilent 1,200 series, Agilent Technologies) of leaf samples from black rot R line SCNU-C-4072 (1-7) and S line SCNU-C-3383 (8-14) of cabbage under different treatments (C, control; 1DM, 1 day mock; 1DT, 1 day treated; 3DM, 3 day mock; 3DT, 3 day treated; 5DM, 5 day mock; 5 DT, 5 day treated) was used to identify individual GSLs . R: Resistant; S: Susceptible.
