# Supplementary material for: Glucosinolate Profile and Glucosinolate Biosynthesis and Breakdown Gene Expression Manifested by Black Rot Disease Infection in Cabbage
Source: Plants (Basel). 2020 Aug 30;9(9):1121. doi: 10.3390/plants9091121 (PMC7569847; doi:10.3390/plants9091121)
Supplement: Supplementary file 1 [file plants-09-01121-s001.zip › Supplementary materials/Supplementary Figures and Tables.docx]

**Supplementary Figures and Tables**

**
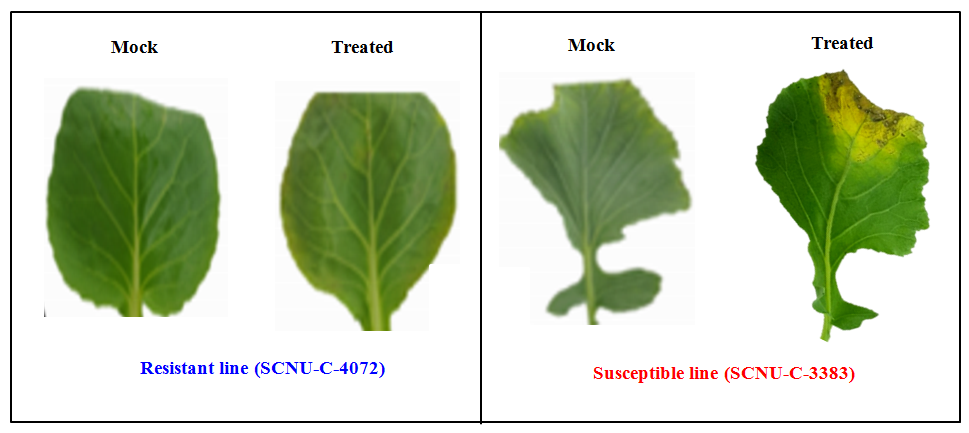
**

**FIGURE S1.** Phenotype screening of selected black rot resistant (SCNU-C-4072) and susceptible (SCNU-C-3383) cabbage lines after inoculation with *X. campestris* pv*. campestris* race 4*.* Photograph was taken at 14 days after inoculation (DAI). Black rot-resistant (R) and -susceptible (S) lines were defined according to the disease scoring scale (0-3).

**
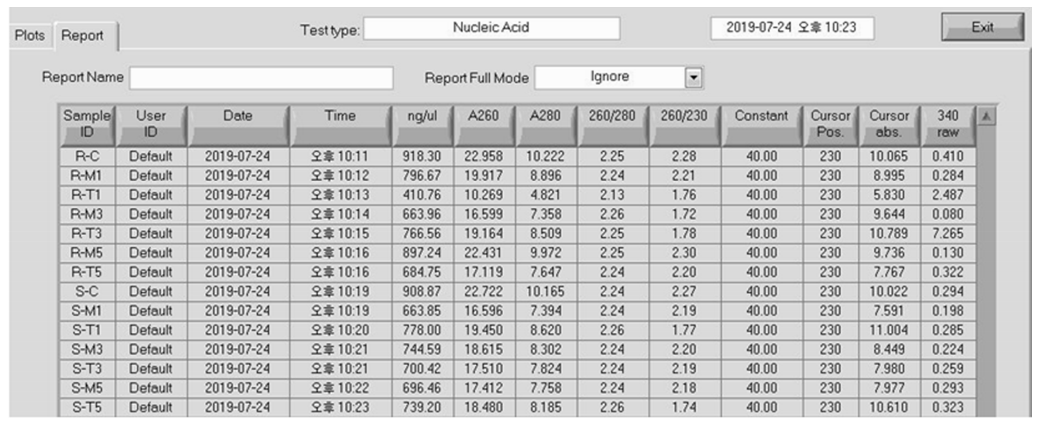
**

**FIGURE S2.** RNA measurement of leaf samples (Black rot) by NanoDrop (ND-1000 Spectrophotometer) (C, control; 1DM, 1 day mock; 1DT, 1 day treated; 3DM, 3 day mock; 3DT, 3 day treated; 5DM, 5 day mock; 5 DT, 5 day treated). R: Resistant; S: Susceptible.

**
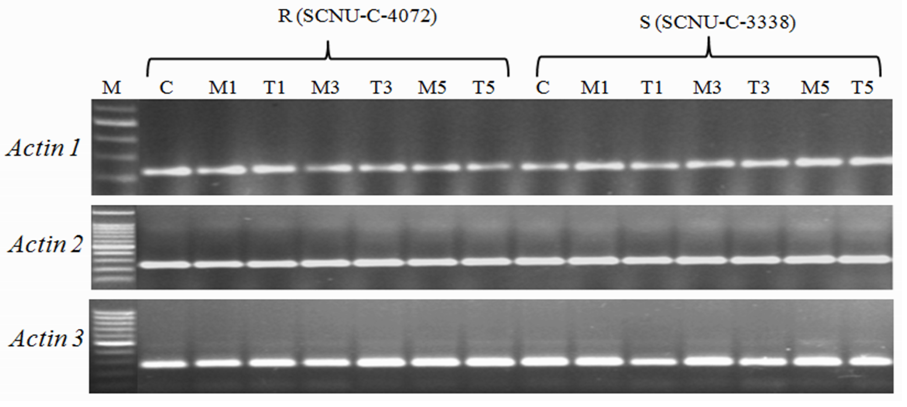
**

**FIGURE S3.** RT-PCR amplification of black rot cDNA sample of cabbage R (SCNU-C-4072) and S (SCNU-C-3383) lines by three *actins* (*actin* 1, 2, and 3) primer of *Brassica oleracea*. (C, control; 1DM, 1 day mock; 1DT, 1 day treated; 3DM, 3 day mock; 3DT, 3 day treated; 5DM, 5 day mock; 5 DT, 5 day treated). R: Resistant; S: Susceptible. M; 100 bp DNA ladder

**
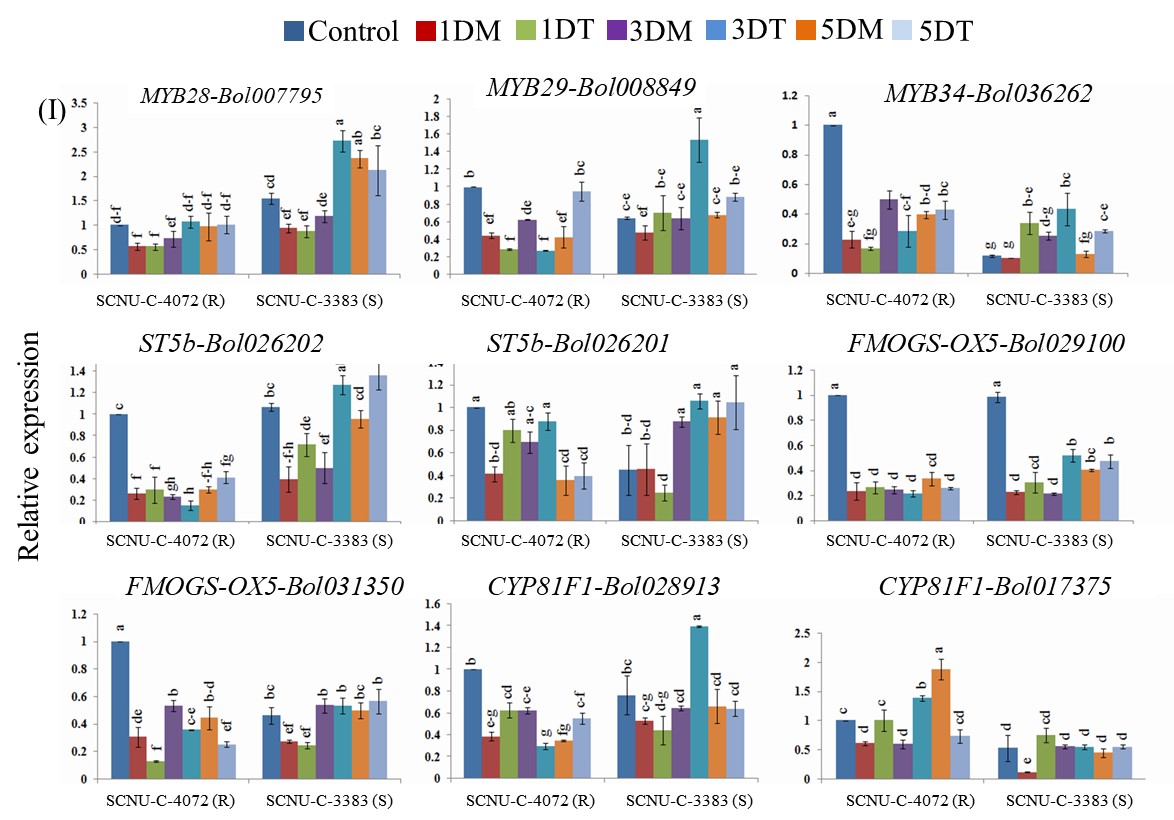
**

**
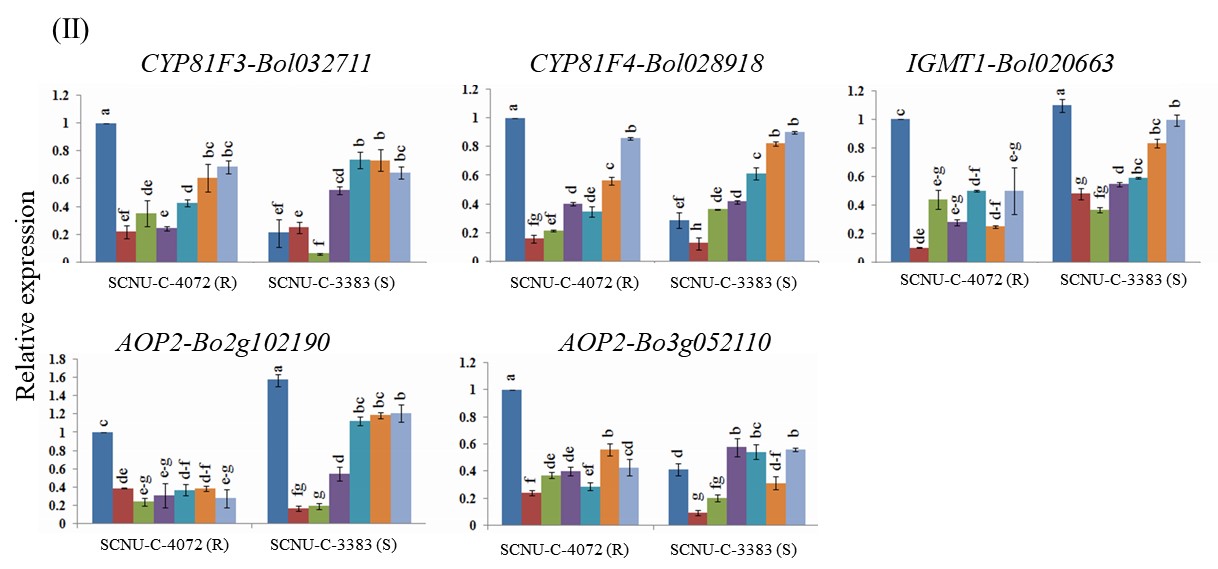
**

**FIGURE S4 (I-II).** Inconsistent response of expression of transcription factor related and glucosinolate biosynthesis genes in black rot R (SCNU-C-4072) and S (SCNU-C-3383) lines of cabbage. C, control; 1DM, 1 day mock; 1DT, 1 day treated; 3DM, 3 day mock; 3DT, 3 day treated; 5DM, 5 day mock; 5 DT, 5 day treated. The mean of three biological replicates are used. Vertical bars indicate standard deviation. Different letters indicate statistically significant differences between R and S lines and treatment interactions. R: Resistant; S: Susceptible.

**TABLE S1.** Phenotypic performance of cabbage (*Brassica oleracea* var. *capitata*) lines after inoculation with *Xcc* race 4 to identify the black rot resistance cabbage lines following by the most popular disease scoring scale based on lesion size produced on the leaves (0-3:0=R, 1=MR, 2=S and 3=HS) (Vicente et al. 2001). Cabbage leaves rated 0 for resistant (R), 1 for moderately resistant (MR), 2 for susceptible (S) and 3 for highly susceptible (HS). Black bold color resistant and susceptible lines were selected for GSL profiling and GSL biosynthesis including with GSL break-down related gene expressions.

| **Sl.** | **Cabbage lines** | **Phenotypic**  **reaction at 14 DAI** | **Sl.** | **Cabbage lines** | **Phenotypic**  **reaction at 14 DAI** |
| --- | --- | --- | --- | --- | --- |
| 1 | SCNU-C-005 | S | 31 | SCNU-C-056 | MR |
| 2 | SCNU-C-007 | S | 32 | SCNU-C-059 | S |
| 3 | SCNU-C-009 | S | 33 | SCNU-C-060 | R |
| 4 | SCNU-C-013 | S | 34 | SCNU-C-061 | MR |
| 5 | SCNU-C-014 | MR | 35 | SCNU-C-062 | S |
| 6 | SCNU-C-016 | MR | 36 | SCNU-C-063 | S |
| 7 | SCNU-C-018 | S | 37 | SCNU-C-064 | R |
| 8 | SCNU-C-019 | MR | 38 | SCNU-C-065 | HS |
| 9 | SCNU-C-020 | S | 39 | SCNU-C-287 | S |
| 10 | SCNU-C-021 | MR | 40 | SCNU-C-289 | S |
| 11 | SCNU-C-024 | S | 41 | SCNU-C-292 | MR |
| 12 | SCNU-C-025 | S | 42 | SCNU-C-293 | S |
| 13 | SCNU-C-027 | MR | 43 | SCNU-C-295 | S |
| 14 | SCNU-C-028 | MR | 44 | SCNU-C-299 | S |
| 15 | SCNU-C-030 | S | 45 | SCNU-C-300 | S |
| 16 | SCNU-C-031 | MR | 46 | SCNU-C-311 | MR |
| 17 | SCNU-C-032 | S | 47 | SCNU-C-314 | MR |
| 18 | SCNU-C-033 | S | 48 | SCNU-C-319 | MR |
| 19 | SCNU-C-035 | S | 49 | SCNU-C-320 | S |
| 20 | SCNU-C-036 | S | 50 | SCNU-C-321 | HS |
| 21 | SCNU-C-040 | S | 51 | SCNU-C-323 | S |
| 22 | SCNU-C-041 | MR | 52 | SCNU-C-325 | S |
| 23 | SCNU-C-043 | S | 53 | SCNU-C-326 | S |
| 24 | SCNU-C-048 | S | 54 | SCNU-C-327 | S |
| 25 | SCNU-C-049 | S | 55 | SCNU-C-345 | MR |
| 26 | SCNU-C-051 | S | 56 | SCNU-C-346 | S |
| 27 | SCNU-C-052 | S | 57 | SCNU-C-347 | S |
| 28 | SCNU-C-053 | S | 58 | **SCNU-C-4072** | **R** |
| 29 | SCNU-C-054 | S | 59 | **SCNU-C-3383** | **HS** |
| 30 | SCNU-C-055 | HS |  |  | |

**TABLE S2.** Primer sequences and efficiency (Abuyusuf et al. 2018; Robin et al. 2016) for the 43 glucosinolate (GSL) biosynthesis and GSL break-down related genes including three *actin* genes (*actin1*, *actin2* and *actin3*) used in the relative expression analysis through q-PCR in black rot resistant (SCNU-C-4072) and susceptible (SCNU-C-3383) lines.

| **Gene  Name** | **Accession  Number** | **cDNA  Size (bp)** | **Forward Primer Sequence** | **Reverse Primer Sequence** | **Product  Size (bp)** |
| --- | --- | --- | --- | --- | --- |
| **Transcription factor-related genes (11 genes)** | | | | | |
| ***MYB28*** | Bol007795 | 558 | CCACACCAGTTCAGAGAGGT | GGGAAATGGATCGAAGTCAGC | 221 |
|  | Bol036286 | 615 | GAAGGTAGCTTGAATGCTAATAC | ATTCATGTAGTGCTCCTCATTC | 249 |
|  | Bol017019 | 426 | GTTGCGGCTAAGGTCACTTCT | CAGAAGTAGCGTTGATCTCATGC | 223 |
|  | Bol036743 | 426 | CTTGGGCGCTGCTACATTAC | ATCGTTCTCCTCGTTGTGGT | 241 |
| ***MYB29*** | Bol008849 | 513 | CGCCCAAGACTTCTGAGTT | TGATATTGCCCATGGAAGCTG | 234 |
| ***MYB34*** | Bol007760 | 843 | TG‍AAGGAGGATGGCGTACTC | CAGTTCGTCCCGCCAAATTA | 203 |
|  | Bol017062 | 951 | AAGGTGGATGGCGTACTCTC | TGTGAGTGGTTGGATCGACA | 279 |
|  | Bol036262 | 294 | ATGTCGACATCTTCGGGTTT | CCAAGAATCAAGAAACTCCA | 222 |
| ***MYB51*** | Bol013207 | 1002 | GGTGAAGAATAGCAACAAGA | TTCGGAGTTAACGGTGACAC | 184 |
|  | Bol030761 | 990 | CGTGGATTACCGGGAAGAAC | TCTTCATTCTTGACCTTCTC | 227 |
| ***MYB122*** | Bol026204 | 981 | GACCATTCCGAGACATTGCC | GCATCGTGGATCATGTGGAG | 284 |
| **Aliphatic biosynthesis-related genes (10 genes)** | | | | | |
| ***ST5b*** | Bol026201 | 1035 | CCGAGCCGTCAGAATTCAAG | GCTATGGCGAAAGTGAGAGC | 247 |
|  | Bol026202 | 1035 | AAGCCTTGACTTTCGCCATC | ACTTCACAACTGAGTCCGGT | 204 |
| ***ST5c*** | Bol030757 | 1014 | CCACGCCCAAAACTTCTTCA | TGAGTGGAGAAGAGCGTGTT | 246 |
| ***FMOGS-OX2*** | Bol010993 | 1386 | GAGAAGGTATCCGAGCCACA | GTCCACTGCAAACAACGACT | 200 |
| ***FMOGS-OX5*** | Bol029100 | 1347 | CTTGCTCCAACGCTTTCCTT | CCTCAGCTCTCCAGTGTTCA | 280 |
|  | Bol031350 | 1380 | ATGGCACCCTCTTGCAGTCC | AGTCGTAGACGCTAGAGTGG | 226 |
| ***AOP2*** | Bo2g102190 | 1104 | GGAACGTGTCTCCAAAACCC | TAGCACCATCACCAGCATCA | 354 |
|  | Bo3g052110 | 948 | ATGGGTTCACACAGTACTCC | GGCCTCAACAGGTAGCTGGA | 216 |
|  | Bo9g006240 | 1032 | ATGGGTGCAGACACTCCTCA | TAGCCTCAACTGGTAACTCG | 214 |
| ***GSL-OH*** | Bol033373 | 243 | GATTGTGCAAAAGGCTTGT | AGAGCATTAGGATTAGGAGGA | 188 |
| **Indolic biosynthesis-related genes (17 genes)** | | | | | |
| ***ST5a*** | Bol026200 | 1017 | GTCCGGTTGCAAGATGGTTT | CCTCTCCGGGTTCTCTTTGT | 214 |
|  | Bol039395 | 1014 | TGCCGTTTGTGAAGAGGTTG | CCCAATCTCCAACCTTCCCT | 210 |
| ***CYP81F4*** | Bol032712 | 1506 | CGGTGGAGGAGAAGGAGAAA | CTGACACATGGCTCGTAACG | 226 |
|  | Bol032714 | 960 | ACCCTGGTGAATACTTGCCA | GAAACACACTGAAGCAGAAC | 239 |
|  | Bol028918 | 1503 | GTTTGCGGCATCAGAGACAT | GAATAGTCCACGCGTTCACC | 299 |
| ***CYP81F1*** | Bol017375 | 369 | AAGCAGAGCGGTTCAAGAAG | GCGTGACCATTGTGTTACCA | 204 |
|  | Bol017376 | 246 | CCGTCTCCTTCAACGGTTCT | CGACGTATTTACCGGTGAGC | 170 |
|  | Bol028913 | 1500 | GAGACCTCCGCAGTAACCTT | GTCCTCCGTCGGTCTTCTAG | 222 |
|  | Bol028914 | 1497 | CTTTCCAACTGACGGCCAAA | CGTTAGGTCCGAGAAAAGCG | 257 |
| ***CYP81F2*** | Bol012237 | 933 | GCAGCCGTGACACTAGAATG | TCCGCCAATCTTGAGGTCTT | 231 |
|  | Bol014239 | 1482 | TTGTACCGCGTTCTCCTTCT | GACACCATCCTCTGACCCAA | 238 |
|  | Bol026044 | 1482 | TCGGCAATCTCCACCTCGTG | GTCGCTCTGACCGGTGAAGC | 158 |
| ***CYP81F3*** | Bol028919 | 1500 | CGAGAAGAAAGTGAAAGCTG | TAAGGCCTTTGATAGTGACG | 164 |
|  | Bol032711 | 1491 | GTGAAAGCTGTTGGAGAAGC | GTTCCGGCGATCATCATGCC | 179 |
| ***IGMT1*** | Bol007029 | 1119 | GTGTTCCTCTCACCTTCCGA | GTGTTGAGGAAGACGCTGTC | 260 |
|  | Bol020663 | 342 | AGATGCCATGATCTTGAAACGT | CCAGCAATGATAAGCCTGACA | 298 |
| ***IGMT2*** | Bol007030 | 1125 | AGCCTTTCCCATGGTTCTCA | TCTCTCGCCCTTTCCAAACT | 223 |
| **Glucosinolate break-down related genes (5 genes)** | | | | | |
| ***PEN2*** | Bol030092 | 1299 | GCATCATCATCCAACAGCGT | ACGCCTTGATCAGTTCTCCA | 207 |
| ***TGG1*** | Bol017328 | 822 | TCTTAACGTGTGGGATGGCT | CCTCCTTTGTTCACTCCCCT | 210 |
| ***TGG2*** | Bol028319 | 1179 | TCGTCTCAACAGTAGCAGCT | AGTAGCGTTGAGTTCGTCCA | 220 |
|  | Bol025706 | 663 | GGTGAGTAGGGGAGTGAACC | TTCCTCGGTGAAGTTGGGAA | 244 |
| ***TGG5*** | Bol031599 | 1326 | CCAGATCACAGTTCCGGAGA | ACTATACGCCGGCTCAAGAA | 293 |
| **House keeping genes** | | | | | |
| ***Actin1*** | AF044573 |  | TTCTCTCTTCCACACGCCAT | CTTGTCCTGCGGGTAATTCG | 235 |
| ***Actin2*** | JQ435879 |  | GTCGCTATTCAAGCTGTTCTCT | GAGAGCTTCTCCTTGATGTCTC | 251 |
| ***Actin3*** | XM_013753 |  | ATCACACTTTCTACAATGAGC | TCGTAGATTGGCACAGTGTGAG | 241 |

**TABLE S3.** Test statistic F and P-values for glucosinolate component in black rot resistant (R) line SCNU-C-4072 and susceptible (S) line SCNU-C-3383 of cabbage at a significance level of α=0.05, respectively.

| **Glucosinolate components** | **R & S lines** | | **R & S lines and treatment interaction** | |
| --- | --- | --- | --- | --- |
|  | **F value** | **P value** | **F value** | **P value** |
| Glucoiberin | 14.68 | <0.01 | 38.25 | <0.01 |
| Progoitrin | 8.38 | 0.006 | 115.14 | <0.01 |
| Sinigrin | 4.75 | 0.035 | 259.63 | <0.01 |
| Gluconapin | 36.83 | <0.01 | 1596.02 | <0.01 |
| Glucoiberverin | 12.28 | 0.001 | 367.64 | <0.01 |
| Glucoerucin | 0.45 | 0.505 | 559.47 | <0.01 |
| 4-Hydroxyglucobrassicin | 13.57 | 0.001 | 4299.18 | <0.01 |
| Glucobrassicin | 18.00 | <0.01 | 993.57 | <0.01 |
| 4-Methoxyglucobrassicin | 15.64 | <0.01 | 329.76 | <0.01 |
| Neoglucobrassicin | 3.11 | 0.086 | 843.57 | <0.01 |

**TABLE S4.** Test statistic F and P-values for expression of glucosinolate biosynthesis and myrosinase genes in black rot resistant (R) line SCNU-C-4072 and susceptible (S) line SCNU-C-3383 of cabbage at a significance level of *P < 0.05*, respectively.

| **Relative expression of genes** | **R and S lines** | | **R and S lines and treatment Interactions** | |
| --- | --- | --- | --- | --- |
|  | **F value** | **P value** | **F value** | **P value** |
| *MYB28-Bol007795* | 25.33 | <0.01 | 35.02 | <0.01 |
| *MYB28-Bol036286* | 2.40 | 0.129 | 140.98 | <0.01 |
| *MYB28-Bol017019* | 3.51 | 0.068 | 312.26 | <0.01 |
| *MYB28-Bol036743* | 0.68 | 0.415 | 112.36 | <0.01 |
| *MYB29-Bol008849* | 5.16 | 0.029 | 29.48 | <0.01 |
| *MYB34-Bol007760* | 11.01 | 0.002 | 905.30 | <0.01 |
| *MYB34-Bol017062* | 3.91 | 0.055 | 215.53 | <0.01 |
| *MYB34-Bol036262* | 8.81 | 0.005 | 52.88 | <0.01 |
| *MYB51-Bol013207* | 9.73 | 0.003 | 140.71 | <0.01 |
| *MYB51-Bol030761* | 8.38 | 0.006 | 3265.96 | <0.01 |
| *MYB122-Bol026204* | 3.08 | 0.087 | 561.17 | <0.01 |
| *ST5a-Bol026200* | 6.28 | 0.016 | 708.00 | <0.01 |
| *ST5a-Bol039395* | 7.81 | 0.008 | 474.53 | <0.01 |
| *ST5b-Bol026202* | 27.03 | <0.01 | 70.06 | <0.01 |
| *ST5b-Bol026201* | 0.59 | 0.445 | 13.89 | <0.01 |
| *ST5c-Bol030757* | 1.54 | 0.221 | 302.86 | <0.01 |
| *FMOGS-OX2-Bol010993* | 3.84 | 0.057 | 71.18 | <0.01 |
| *FMOGS-OX5-Bol029100* | 1.03 | 0.317 | 115.00 | <0.01 |
| *FMOGS-OX5-Bol031350* | 0.04 | 0.842 | 53.50 | <0.01 |
| *GSL-OH-Bol033373* | 9.28 | 0.004 | 920.76 | <0.01 |
| *CYP81F4-Bol032712* | 1.17 | 0.286 | 1618.49 | <0.01 |
| *CYP81F4-Bol032714* | 1.62 | 0.211 | 372.12 | <0.01 |
| *CYP81F4-Bol028918* | 0.00 | 0.988 | 347.36 | <0.01 |
| *CYP81F1-Bol017375* | 24.10 | <0.01 | 48.57 | <0.01 |
| *CYP81F1-Bol017376* | 5.83 | 0.020 | 534.47 | <0.01 |
| *CYP81F1-Bol028913* | 4.44 | 0.041 | 37.60 | <0.01 |
| *CYP81F1-Bol028914* | 3.53 | 0.067 | 337.58 | <0.01 |
| *CYP81F2-Bol012237* | 0.02 | 0.888 | 872.76 | <0.01 |
| *CYP81F2-Bol014239* | 5.12 | 0.029 | 601.54 | <0.01 |
| *CYP81F2-Bol026044* | 7.02 | 0.011 | 216.78 | <0.01 |
| *CYP81F3-Bol028919* | 5.18 | 0.028 | 1282.05 | <0.01 |
| *CYP81F3-Bol032711* | 0.43 | 0.516 | 63.88 | <0.01 |
| *IGMT1-Bol007029* | 6.34 | 0.016 | 1158.99 | <0.01 |
| *IGMT1-Bol020663* | 9.76 | 0.003 | 98.85 | <0.01 |
| *IGMT2-Bol007030* | 6.50 | 0.015 | 1166.92 | <0.01 |
| *AOP2-Bo2g102190* | 11.46 | 0.002 | 156.52 | <0.01 |
| *AOP2-Bo3g052110* | 1.56 | 0.219 | 97.16 | <0.01 |
| *AOP2-Bo9g006240* | 3.87 | 0.056 | 947.92 | <0.01 |
| *PEN2-Bol030092* | 5.11 | 0.029 | 308.44 | <0.01 |
| *TGG1-Bol017328* | 19.08 | <0.01 | 61.16 | <0.01 |
| *TGG2-Bol028319* | 0.32 | 0.576 | 32.54 | <0.01 |
| *TGG2-Bol025706* | 6.05 | 0.018 | 16.43 | <0.01 |
| *TGG5-Bol031599* | 13.61 | 0.001 | 20.17 | <0.01 |

**TABLE S5**. Heat maps comparing black rot resistant (SCNU-C-4072) and susceptible SCNU-C-3383) lines shows fold changes in individual glucosinolate component in *X.campestris* pv. *campestris*–inoculated leaf samples compared to respective mock-treated samples.1 Day, T1/M1; 3 Day, T3/M3, 5 Day, T5/M5; M1, 1day mock; T1, 1day treated; M3, 3 day mock; T3, 3 day treated;M5, 5 day mock; T5, 5 day treated.

| **Glucosinolate component** | **Treatments** | **SCNU-C-4072** | **SCNU-C-3383** |
| --- | --- | --- | --- |
| Glucoiberin | 1 Day | 1.149 | 0.933 |
|  | 3 Day | 1.374 | 0.958 |
|  | 5 Day | 0.727 | 0.378 |
| Progoitrin | 1 Day | 0.500 | 1.000 |
|  | 3 Day | 1.563 | 1.571 |
|  | 5 Day | 4.000 | 1.333 |
| Sinigrin | 1 Day | 4.080 | 0.682 |
|  | 3 Day | 1.535 | 0.784 |
|  | 5 Day | 1.003 | 1.008 |
| Gluconapin | 1 Day | 4.796 |  |
|  | 3 Day | 1.971 |  |
|  | 5 Day | 1.198 |  |
| Glucoiberverin | 1 Day | 3.659 | 1.324 |
|  | 3 Day | 5.191 | 1.221 |
|  | 5 Day | 1.047 | 0.743 |
| Glucoerucin | 1 Day | 5.441 | 1.645 |
|  | 3 Day | 1.837 | 1.183 |
|  | 5 Day | 1.252 | 1.135 |
| 4-Hydroxyglucobrassicin | 1 Day | 5.789 |  |
|  | 3 Day | 10.271 |  |
|  | 5 Day | 1.000 |  |
| Glucobrassicin | 1 Day | 5.205 | 3.281 |
|  | 3 Day | 1.412 | 0.980 |
|  | 5 Day | 1.051 | 0.783 |
| 4-Methoxyglucobrassicin | 1 Day | 2.895 | 1.558 |
|  | 3 Day | 4.691 | 1.020 |
|  | 5 Day | 1.065 | 1.547 |
| Neoglucobrassicin | 1 Day | 1.717 | 1.378 |
|  | 3 Day | 3.613 | 1.284 |
|  | 5 Day | 0.966 | 0.998 |

**TABLE S6.** Heat maps comparing black rot resistant line SCNU-C-4072 and susceptible line SCNU-C-3383 shows fold changes in expression of transcription factor related genes in *X. campestris* pv. *campestris*-inoculated leaf samples compared to respective mock-treated samples. 1 Day, T1/M1; 3 Day, T3/M3, 5Day, T5/M5; M1, 1day mock; T1, 1day treated; M3, 3 day mock; T3, 3 day treated;M5, 5 day mock; T5, 5 day treated.

| **Genes (Transcription factors)** | **Treatments** | **SCNU-C-4072** | **SCNU-C-3383** |
| --- | --- | --- | --- |
| *MYB28-Bol007795* | 1 Day | 0.984 | 0.938 |
|  | 3 Day | 1.473 | 2.302 |
|  | 5 Day | 1.046 | 0.899 |
| *MYB28-Bol036286* | 1 Day | 1.068 | 1.324 |
|  | 3 Day | 1.316 | 11.185 |
|  | 5 Day | 1.297 | 1.272 |
| *MYB28-Bol017019* | 1 Day | 4.749 | 10.387 |
|  | 3 Day | 0.827 | 1.803 |
|  | 5 Day | 0.852 | 1.097 |
| *MYB28-Bol036743* | 1 Day | 2.765 | 8.945 |
|  | 3 Day | 0.781 | 2.117 |
|  | 5 Day | 1.001 | 0.876 |
| *MYB29-Bol008849* | 1 Day | 0.648 | 1.468 |
|  | 3 Day | 0.435 | 2.394 |
|  | 5 Day | 2.220 | 1.294 |
| *MYB34-Bol017062* | 1 Day | 36.851 | 0.919 |
|  | 3 Day | 2.426 | 1.103 |
|  | 5 Day | 1.250 | 1.146 |
| *MYB34-Bol007760* | 1 Day | 1.099 | 8.996 |
|  | 3 Day | 0.826 | 2.559 |
|  | 5 Day | 1.001 | 1.373 |
| *MYB34-Bol036262* | 1 Day | 0.732 | 3.280 |
|  | 3 Day | 0.573 | 1.704 |
|  | 5 Day | 1.081 | 2.201 |
| *MYB51-Bol013207* | 1 Day | 1.444 | 1.559 |
|  | 3 Day | 1.206 | 5.417 |
|  | 5 Day | 0.922 | 1.174 |
| *MYB51-Bol030761* | 1 Day | 0.901 | 1.417 |
|  | 3 Day | 1.652 | 9.612 |
|  | 5 Day | 0.620 | 1.336 |
| *MYB122-Bol026204* | 1 Day | 0.449 | 3.594 |
|  | 3 Day | 49.404 | 0.453 |
|  | 5 Day | 0.222 | 1.313 |

**TABLE S7.** Heat maps comparing black rot resistant line SCNU-C-4072 and susceptible line SCNU-C-3383 shows fold changes in expression of aliphatic glucosinolate biosynthesis genes in *X. campestris* pv. *campestris*-inoculated leaf samples compared to respective mock-treated samples. 1 Day, T1/M1; 3 Day, T3/M3, 5 Day, T5/M5; M1, 1day mock; T1, 1day treated; M3, 3 day mock; T3, 3day treated; M5, 5 day mock; T5, 5 day treated.

| **Genes (Aliphatic)** | **Treatments** | **SCNU-C-4072** | **SCNU-C-3383** |
| --- | --- | --- | --- |
| *ST5b-Bol026202* | 1 Day | 1.129 | 1.814 |
|  | 3 Day | 0.658 | 2.530 |
|  | 5 Day | 1.364 | 1.424 |
| *ST5b-Bol026201* | 1 Day | 1.920 | 0.547 |
|  | 3 Day | 1.270 | 1.208 |
|  | 5 Day | 1.110 | 1.143 |
| *GSL-OH-Bol033373* | 1 Day | 0.984 | 1.456 |
|  | 3 Day | 1.087 | 6.292 |
|  | 5 Day | 0.748 | 1.247 |
| *ST5c-Bol030757* | 1 Day | 1.134 | 1.193 |
|  | 3 Day | 13.121 | 3.062 |
|  | 5 Day | 0.708 | 1.286 |
| *FMOGS-OX2-Bol010993* | 1 Day | 4.009 | 7.977 |
|  | 3 Day | 1.526 | 1.267 |
|  | 5 Day | 1.023 | 0.991 |
| *FMOGS-OX5-Bol029100* | 1 Day | 1.113 | 1.342 |
|  | 3 Day | 0.867 | 2.398 |
|  | 5 Day | 0.770 | 1.169 |
| *FMOGS-OX5-Bol031350* | 1 Day | 0.426 | 0.893 |
|  | 3 Day | 0.669 | 0.997 |
|  | 5 Day | 0.564 | 1.136 |
| *AOP2-Bo2g102190* | 1 Day | 0.624 | 1.152 |
|  | 3 Day | 1.191 | 2.056 |
|  | 5 Day | 0.730 | 1.019 |
| *AOP2-Bo3g052110* | 1 Day | 1.544 | 2.067 |
|  | 3 Day | 0.718 | 0.944 |
|  | 5 Day | 0.765 | 1.789 |
| *AOP2-Bo9g006240* | 1 Day | 10.304 | 0.445 |
|  | 3 Day | 1.174 | 1.001 |
|  | 5 Day | 0.828 | 1.239 |

**TABLE S8.** Heat maps comparing black rot resistant line SCNU-C-4072 and susceptible line SCNU-C-3383 shows fold changes in expression of indolic glucosinolate biosynthesis genes in *X. campestris* pv. *campestris*-inoculated leaf samples compared to respective mock-treated samples. 1 Day, T1/M1; 3 Day, T3/M3, 5 Day, T5/M5; M1, 1day mock; T1, 1day treated; M3, 3 day mock; T3, 3 day treated; M5, 5 day mock; T5, 5 day treated.

| **Genes (Indolic)** | **Treatments** | **SCNU-C-4072** | **SCNU-C-3383** |
| --- | --- | --- | --- |
| *ST5a-Bol039395* | 1 Day | 0.958 | 1.938 |
|  | 3 Day | 1.267 | 6.223 |
|  | 5 Day | 1.117 | 1.210 |
| *ST5a-Bol026200* | 1 Day | 0.921 | 2.368 |
|  | 3 Day | 2.011 | 15.034 |
|  | 5 Day | 0.393 | 1.221 |
| *CYP81F1-Bol028913* | 1 Day | 1.627 | 0.834 |
|  | 3 Day | 0.477 | 2.160 |
|  | 5 Day | 1.581 | 0.970 |
| *CYP81F1-Bol028914* | 1 Day | 1.176 | 0.872 |
|  | 3 Day | 2.324 | 38.203 |
|  | 5 Day | 4.564 | 3.042 |
| *CYP81F1-Bol017375* | 1 Day | 1.644 | 6.599 |
|  | 3 Day | 2.345 | 0.986 |
|  | 5 Day | 0.391 | 1.230 |
| *CYP81F1-Bol017376* | 1 Day | 11.862 | 0.760 |
|  | 3 Day | 3.492 | 0.840 |
|  | 5 Day | 1.442 | 1.228 |
| *CYP81F2-Bol012237* | 1 Day | 4.883 | 1.917 |
|  | 3 Day | 6.407 | 1.857 |
|  | 5 Day | 0.788 | 0.944 |
| *CYP81F2-Bol014239* | 1 Day | 0.641 | 2.225 |
|  | 3 Day | 2.653 | 4.536 |
|  | 5 Day | 0.754 | 1.279 |
| *CYP81F2-Bol026044* | 1 Day | 3.615 | 1.228 |
|  | 3 Day | 0.786 | 12.047 |
|  | 5 Day | 1.237 | 1.186 |
| *CYP81F3-Bol032711* | 1 Day | 1.600 | 0.244 |
|  | 3 Day | 1.757 | 1.421 |
|  | 5 Day | 1.127 | 0.882 |
| *CYP81F3-Bol028919* | 1 Day | 1.940 | 1.806 |
|  | 3 Day | 0.659 | 14.417 |
|  | 5 Day | 0.969 | 1.288 |
| *CYP81F4-Bol032712* | 1 Day | 1.621 | 1.681 |
|  | 3 Day | 9.691 | 7.007 |
|  | 5 Day | 1.340 | 1.271 |
| *CYP81F4-Bol032714* | 1 Day | 4.159 | 1.353 |
|  | 3 Day | 25.291 | 4.102 |
|  | 5 Day | 0.973 | 1.202 |
| *CYP81F4-Bol028918* | 1 Day | 1.359 | 2.912 |
|  | 3 Day | 0.869 | 1.478 |
|  | 5 Day | 1.525 | 1.098 |
| *IGMT1-Bol007029* | 1 Day | 1.151 | 1.529 |
|  | 3 Day | 3.095 | 8.880 |
|  | 5 Day | 0.690 | 1.279 |
| *IGMT1-Bol020663* | 1 Day | 4.433 | 0.764 |
|  | 3 Day | 1.795 | 1.085 |
|  | 5 Day | 2.013 | 1.193 |
| *IGMT2-Bol007030* | 1 Day | 1.649 | 1.651 |
|  | 3 Day | 2.810 | 11.439 |
|  | 5 Day | 0.930 | 1.306 |

**TABLE S9.** Heat maps comparing black rot resistant line SCNU-C-4072 and susceptible line SCNU-C-3383 shows fold changes in expression of glucosinolate break-down related genes in *X. campestris* pv. *campestris*-inoculated leaf samples compared to respective mock-treated samples. 1 Day, T1/M1; 3 Day, T3/M3, 5 Day, T5/M5; M1, 1day mock; T1, 1day treated; M3, 3 day mock; T3, 3 day treated; M5, 5 day mock; T5, 5 day treated.

| **Genes (Break-down related)** | **Treatments** | **SCNU-C-4072** | **SCNU-C-3383** |
| --- | --- | --- | --- |
| *PEN2-Bol030092* | 1 Day | 8.470 | 1.348 |
|  | 3 Day | 1.414 | 1.275 |
|  | 5 Day | 1.357 | 1.041 |
| *TGG1-Bol017328* | 1 Day | 0.915 | 0.872 |
|  | 3 Day | 0.285 | 1.271 |
|  | 5 Day | 2.099 | 1.213 |
| *TGG2-Bol028319* | 1 Day | 0.469 | 0.346 |
|  | 3 Day | 0.552 | 0.442 |
|  | 5 Day | 0.712 | 1.171 |
| *TGG2-Bol025706* | 1 Day | 1.411 | 4.146 |
|  | 3 Day | 1.068 | 1.463 |
|  | 5 Day | 1.670 | 1.405 |
| *TGG5-Bol031599* | 1 Day | 1.551 | 0.840 |
|  | 3 Day | 0.877 | 1.795 |
|  | 5 Day | 0.852 | 1.305 |

**TABLE S10.** Component loadings of cabbage black rot resistant (R;SCNU-C-4072) and susceptible (S;SCNU-C-3383) lines, glucosinolate compounds and glucosinolate biosynthesis and GSL break-down related gene responses as determined by the principal component analysis (PCA) Tukey’s test was done based on P<0.05.

| **Variable** | **PC1** | **PC2** | **PC3** | **PC4** |
| --- | --- | --- | --- | --- |
| *MYB28 - Bol007795* | 0.197 | -0.064 | -0.026 | -0.014 |
| *MYB28 - Bol036286* | 0.174 | -0.177 | 0.053 | 0.014 |
| *MYB28 - Bol017019* | -0.016 | -0.006 | 0.26 | -0.002 |
| *MYB28-Bol036743* | -0.034 | -0.018 | 0.25 | 0.016 |
| *MYB29 - Bol008849* | 0.215 | -0.019 | -0.004 | 0.049 |
| *MYB34 - Bol017062* | -0.111 | -0.139 | 0.026 | 0.287 |
| *MYB34 - Bol007760* | 0.062 | -0.007 | 0.25 | -0.08 |
| *MYB34 - Bol036262* | 0.054 | 0.021 | -0.168 | 0.039 |
| *MYB51 - Bol013207* | 0.189 | -0.156 | 0.092 | 0.015 |
| *MYB51 - Bol030761* | 0.198 | -0.139 | 0.063 | 0.033 |
| *MYB122-Bol026204* | -0.075 | -0.171 | -0.149 | -0.228 |
| *ST5a - Bol039395* | 0.195 | -0.147 | 0.071 | 0.021 |
| *ST5a - Bol026200* | 0.195 | -0.153 | 0.046 | 0.031 |
| *ST5b - Bol026202* | 0.195 | 0.029 | -0.027 | 0.074 |
| *ST5b - Bol026201* | 0.096 | -0.119 | -0.188 | 0.096 |
| *ST5c - Bol030757* | -0.036 | -0.206 | -0.134 | -0.219 |
| *FMOGS-OX2 - Bol010993* | -0.008 | -0.018 | 0.251 | -0.055 |
| *FMOGS-OX5- Bol029100* | 0.106 | 0.08 | -0.172 | 0.1 |
| *FMOGS-OX5 - Bol031350* | 0.121 | 0.079 | -0.243 | 0.012 |
| *GSL-OH - Bol033373* | 0.203 | -0.135 | 0.059 | 0.034 |
| *CYP81F1 - Bol028913* | 0.194 | -0.051 | -0.061 | 0.171 |
| *CYP81F1 - Bol028914* | 0.19 | -0.151 | 0.024 | 0.05 |
| *CYP81F1 - Bol017375* | -0.093 | -0.102 | -0.104 | -0.034 |
| *CYP81F1 - Bol017376* | -0.107 | -0.136 | -0.007 | 0.297 |
| *CYP81F2 - Bol012237* | -0.035 | -0.188 | -0.071 | -0.244 |
| *CYP81F2 - Bol014239* | 0.191 | -0.159 | 0.016 | 0.003 |
| *CYP81F2 - Bol026044* | 0.197 | -0.151 | 0.032 | 0.042 |
| *CYP81F3 - Bol032711* | 0.108 | -0.029 | -0.222 | 0.079 |
| *CYP81F3 - Bol028919* | 0.191 | -0.155 | 0.048 | 0.039 |
| *CYP81F4 - Bol032712* | -0.003 | -0.23 | -0.111 | -0.208 |
| *CYP81F4 - Bol032714* | -0.035 | -0.217 | -0.121 | -0.214 |
| *CYP81F4 - Bol028918* | 0.111 | 0.031 | -0.155 | 0.032 |
| *IGMT1 -Bol007029* | 0.185 | -0.176 | 0.048 | -0.004 |
| *IGMT1-Bol020663* | 0.106 | 0.053 | -0.169 | 0.071 |
| *IGMT2 - Bol007030* | 0.182 | -0.18 | 0.057 | -0.025 |
| *AOP2-Bo2g102190* | 0.157 | 0.036 | -0.141 | 0.055 |
| *AOP2-Bo3g052110* | 0.086 | 0.029 | -0.231 | 0.131 |
| *AOP2-Bo9g006240* | -0.107 | -0.107 | 0.025 | 0.314 |
| *TGG1-Bol017328* | 0.129 | 0.142 | -0.06 | 0.064 |
| *TGG2-Bol025706* | 0.015 | -0.034 | 0.285 | -0.097 |
| *PEN2-Bol030092* | -0.117 | -0.14 | 0.045 | 0.28 |
| *TGG2-Bol028319* | 0.038 | 0.199 | -0.208 | -0.008 |
| *TGG5-Bol031599* | 0.113 | 0.06 | -0.06 | 0.078 |
| Glucoiberin (GIB) | -0.088 | -0.062 | -0.198 | 0.052 |
| Progoitrin (PRO) | 0.009 | 0.041 | -0.228 | 0.054 |
| Sinigrin (SIN) | -0.126 | -0.156 | -0.017 | 0.235 |
| Gluconapin (GNA) | -0.147 | -0.109 | -0.037 | 0.245 |
| Glucoiberverin (GIV) | -0.141 | -0.222 | -0.064 | -0.079 |
| 4-Hydroxyglucobrassicin (HGBS) | -0.134 | -0.209 | -0.118 | -0.086 |
| Glucoerucin (GER) | -0.091 | -0.175 | 0.074 | 0.221 |
| Glucobrassicin (GBS) | -0.17 | -0.13 | 0.051 | 0.181 |
| 4-Methoxyglucobrassicin (MGBS) | -0.146 | -0.199 | -0.079 | -0.105 |
| Neoglucobrassicin (NGBS) | -0.072 | -0.219 | -0.111 | -0.178 |
| Total GSL | -0.16 | -0.196 | -0.03 | 0.105 |
| **% Contribution** | **32.2** | **21.0** | **14.0** | **12.0** |
| **Cabbage lines** | **Mean PC scores (±SD)** | | | |
| R-line SCNU-C-4072 | -2.40±2.85b | -0.55±3.72 | -1.06±2.57 | 0.27±3.58 |
| S-line SCNU-C-3383 | 2.40±4.11a | 0.55±3.17 | 1.06±2.75 | -0.27±0.942 |
